# The predominantly selfing plant *Arabidopsis thaliana *experienced a recent reduction in transposable element abundance compared to its outcrossing relative *Arabidopsis lyrata*

**DOI:** 10.1186/1759-8753-3-2

**Published:** 2012-02-07

**Authors:** Nicole de la Chaux, Takashi Tsuchimatsu, Kentaro K Shimizu, Andreas Wagner

**Affiliations:** 1 Molecular Evolution and Evolutionary Systems Biology, Institute of Evolutionary Biology and Environmental Studies, University of Zurich, Zurich, Switzerland; 2 Evolutionary Systems Biology, The Swiss Institute of Bioinformatics, Basel, Switzerland; 3 Evolutionary Functional Genomics, Institute of Plant Biology and Zurich-Basel Plant Science Center, University of Zurich, Zurich, Switzerland; 4 Evolutionary and Ecological Genomics, Institute of Evolutionary Biology and Environmental Studies, University of Zurich, Zurich, Switzerland; 5 Gregor Mendel Institute, Austrian Academy of Sciences, Vienna, Austria; 6 The Santa Fe Institute, Santa Fe, NM, USA

**Keywords:** transposable elements, *Arabidopsis*, mating system, evolutionary dynamics

## Abstract

**Background:**

Transposable elements (TEs) are major contributors to genome evolution. One factor that influences their evolutionary dynamics is whether their host reproduces through selfing or through outcrossing. According to the recombinational spreading hypothesis, for instance, TEs can spread more easily in outcrossing species through recombination, and should thus be less abundant in selfing species. We here studied the distribution and evolutionary dynamics of TE families in the predominantly selfing plant *Arabidopsis thaliana *and its close outcrossing relative *Arabidopsis lyrata *on a genome-wide scale. We characterized differences in TE abundance between them and asked which, if any, existing hypotheses about TE abundances may explain these differences.

**Results:**

We identified 1,819 TE families representing all known classes of TEs in both species, and found three times more copies in the outcrossing *A. lyrata *than in the predominantly selfing *A. thaliana*, as well as ten times more TE families unique to *A. lyrata*. On average, elements in *A. lyrata *are younger than elements in *A*. *thaliana*. In particular, *A. thaliana *shows a marked decrease in element number that occurred during the most recent 10% of the time interval since *A. thaliana *split from *A. lyrata*. This most recent period in the evolution of *A. thaliana *started approximately 500,000 years ago, assuming a splitting time of 5 million years ago, and coincides with the time at which predominant selfing originated.

**Conclusions:**

Our results indicate that the mating system may be important for determining TE copy number, and that selfing species are likely to have fewer TEs.

## Background

Transposable elements (TEs) are major contributors to genome evolution. They can replicate in a genome and therefore create genetic variation on a much larger scale than individual nucleotide changes [1]. Almost all eukaryotic genomes contain TEs but their TE content varies widely among genomes. An exception are many unicellular eukaryotic genomes which lack TEs [2]. TEs mainly use two different intermediates for their replication. Retrotransposons (class I elements) use an RNA intermediate for replication, whereas DNA transposons (class II elements) use a DNA intermediate [1]. Retrotransposons can be further subdivided into long terminal repeat (LTR) and non-LTR elements, based on the presence or absence of LTR sequences in the element.

The evolutionary factors that influence TE abundance have received considerable attention [3-6]. One such factor is life history, such as whether a plant is a perennial or an annual [7-9]. Especially in weedy annuals, for example, selection may favor small genomes to reduce development time. This may indirectly lead to the elimination of TEs, because such elements may be more dispensable than other genomic DNA, especially protein coding genes. Another factor may be effective population size, because selection is less efficient in eliminating deleterious TEs in small populations [8,10,11]. Yet another factor is the mating system [12-14]. More specifically, whether a sexually reproducing species reproduces mostly through inbreeding or through outcrossing may strongly influence TE abundance [15-17]. Theoretical predictions regarding the influence of inbreeding exist, but empirical validation of these predictions has led to conflicting results [14,18,19].

Two main factors can influence TE abundance in selfing and outcrossing species. The first factor is the rate of ectopic recombination between different TE copies. Ectopic recombination is the unequal crossing-over between TEs or any other repetitive sequences at non-homologous chromosome positions. It often results in harmful chromosomal rearrangements [14], and can lead to both insertions and deletions of large chromosomal regions, including many TEs. TEs in selfing species are more likely to be homozygous than TEs in an outcrossing species [12], i.e., TEs in a selfing species more often have an allelic partner at the same chromosomal position than TEs in an outcrossing species. This may reduce ectopic recombination among TE copies, because the two allelic partners can be paired in meiosis and undergo homologous recombination. They are thus less likely to engage in non-homologous pairing and ectopic recombination [12,20]. Whether the reduction of ectopic recombination in inbreeding species would lead to an increase or to a decrease in TE abundance is not clear. Recombination itself is equally likely to increase or decrease copy numbers, but subsequent natural selection may cause a net increase or decrease, depending on whether DNA insertions or deletions are more likely to be deleterious. Both results of population genetic modeling and existing empirical data are equivocal about whether an increase or decrease in TE abundance would occur in selfing populations [14,17,21].

The second major factor is a recombination between individuals of a population [16]. In an outcrossing species, new TEs have the opportunity to spread rapidly through the population by recombination via sexual reproduction. In consequence, new copies can spread even if they have (mildly) deleterious effects. In contrast, in selfing species, recombination is not effective in spreading TEs. New copies are therefore lost by genetic drift and/or purifying selection, and the probability of TE fixation is reduced. This would result in a lower copy number of new TEs in a selfing species. Pertinent empirical data are very limited and based on analysis of different TE families in different species. For example, Schaack *et al*. [17] provided support for the recombinational spreading hypothesis based on six families of DNA elements in the aquatic microcrustacean *Daphnia pulex*, a species that became selfing only recently. In contrast, Dolgin *et al*. [21] provided support against the hypothesis based on the *Tc1 *-like TE family in the selfing nematode *Caenorhabditis elegans *and its outcrossing relative *C. remanei*. We note that existing empirical studies that speak to the effect of inbreeding on TE abundance are based on few TE families. No pertinent genome-scale analysis of TEs in closely related selfing and outcrossing species has been available until recently.

Such a genome-scale analysis has become possible now that the complete genome sequences of two closely related flowering plant species, *Arabidopsis thaliana *(strain Col-0) and *Arabidopsis lyrata *(strain MN47), have become available [22,23]. *A. thaliana *is a self-compatible, predominantly selfing plant with an outcrossing rate estimated at approximately 1 to 3% [24-26]. It has a compact genome of 125 Mb [22]. In contrast, *A. lyrata *is a typically outcrossing species with a genome size exceeding 200 Mb, in which selfing is prevented by the self-incompatibility recognition system controlled by the female and male recognition genes [S-receptor kinase (*SRK *), and S-locus cysteine-rich protein (*SCR*), also known as S-locus protein 11 (*SP11 *), respectively] at the *S*-locus [27-30]. Based on the fossil record and the divergence computed from synonymous substitution rates in the family Brassicaceae at the Chalcone synthase loci (1.0 × 10^-8 ^- 2.0 × 10^-8 ^substitutions per site per year) and Alcohol dehydrogenase loci (9.9 × 10^-9 ^- 2.1 × 10^-8 ^substitutions per site per year), the split between *A. thaliana *and other Arabidopsis species including *A. lyrata *occurred 3.1 to 8.3 million years ago (Mya, 95% confidence limit, mean 5.1 Mya) and 3.3 to 9.0 Mya (mean 5.4 Mya), respectively [31]. Other estimates such as 4.2 to 10.9 Mya incorporating data across diverse plants [32,33], 8.7 ± 1.0, 17.9 ± 4.8 Mya using mutation accumulation lines [34], and 8 to 17.9 Mya based on fossil evidence [35] have also been reported. For our analysis, the splitting time estimation from Koch *et al*. [31] (approx. 5 Mya), which has been commonly used, is most appropriate. The reason is that both the substitution rate and the estimate of when self-compatibility arose in *A. thaliana *are based on this splitting time [36]. We note that the age and abundance distribution of TEs relative to these times, and not their absolute values, are most relevant for this study.

The spread of predominant selfing by the loss of self-incompatibility in *A. thaliana *occurred much more recently than the speciation event between *A. thaliana *and *A. lyrata *[37]. Three lines of pertinent evidence exist. First, the female recognition gene *SRK *has been under purifying selection, and was likely functional until very recently [36]. The data suggest that *A. thaliana *has been self-incompatible for at least 91% of the time since its speciation. Using the splitting time of 5 Mya, the loss of self-incompatibility was estimated to have occurred 0 to 413,000 years ago [36]. Second, all components of the self-incompatibility system except for *SCR/SP11 *still retain functional alleles, suggesting that self-incompatibility was functional until recently [38,39]. Third, alleles at the self-incompatibility locus differ strongly in their polymorphism pattern from the remainder of the genome [39]. It is not clear if some ancestor of extant *A*. *thaliana *was self-incompatible or capable of partial selfing before predominant selfing evolved [39,40]. TEs comprise approximately 10% of the *A. thaliana *genome, and are widely studied [41]. In contrast, TEs in *A. lyrata *are poorly characterized. Because of their close relatedness and because of the likely recent transition between mating systems, these two *Arabidopsis *species are ideal to study the influence selfing might have on TE dynamics and abundance. Studies conducted prior to the completion of the *A. lyrata *genome sequence addressed the question if and how the mating system influences the dynamics of TEs in predominantly selfing *A. thaliana *and outcrossing *A. lyrata*. The results show either no significant difference in copy number, or are consistent with reduced selection due to less ectopic recombination [18,19]. Studies like these focused only on one or a few TE families present in *A. thaliana*, and compared their copy numbers to TEs in *A. lyrata*. Such studies are subject to two important biases. First, they consider few of the TE families present in *A. thaliana*; second, they do not take into account families that may only be present in *A. lyrata*.

Here, we use the genome sequences of strain Col-0 of *A. thaliana *and strain MN47 of *A. lyrata *to identify novel TE families in both genomes, and to compare all TE numbers and similarities in each family among genomes. Although it has been reported very recently that copy numbers differ among *A. thaliana *and *A*. *lyrata *[23,42], we here present a more detailed analysis of TE distributions and ages. We show that the age distribution of TEs in *A. thaliana *points to selfing as an important cause of reduced TE numbers in this species. Our observations are consistent with the recombinational spreading hypothesis, but we cannot exclude a contribution of ectopic recombination to differences in TE abundances.

## Results

### ***A. lyrata *harbors many more TEs**

The best way to identify TEs would be a homology search based on an existing and complete library of elements as query sequences. Unfortunately, even for well-studied organism such as *A. thaliana*, such a library does not exist. First, known TEs may not comprise all TEs in the genome. Even worse, for most species no or only few elements are known. One such species is *A. lyrata*. To alleviate these problems, we used existing information about TEs in our two study genomes, homology searches, and *de novo *identification of elements to identify all TEs in *A. thaliana *[22] and *A. lyrata *[23] (see 'Methods' section for details). After we finished our analysis, results of a similar TE identification process in the two *Arabidopsis *species were published [23,42]. Our study identified similar numbers of TEs in both species, and we briefly compare these numbers with the results of Hollister *et al*. [42] and Hu *et al*. [23] in the 'Discussion' section. In a first step of our analysis, we combined 357 canonical TE sequences, which are prototypic sequences that either represent consensus sequences or a sequence example for a TE family, for *A. thaliana *from Repbase Update [43] with TE families we identified in a *de novo *search in both genomes using RepeatScout [44] (see 'Methods' section). Excluding redundant element families, this approach identified 1,819 different TE families in the two genomes. The majority were DNA transposons (822 families in total), followed by LTR elements (678 families), non-LTR elements (143 families), and 176 families that were not classifiable unambiguously. From here on, we will refer to families derived from Repbase Update as RUxxxx; we will refer to our newly identified families using RepeatScout as RSxxxx, where xxxx stands for a one- to four-digit-long identifier, e.g., RU287. For families from Repbase Update, we will additionally list the name of the family as used in Repbase Update. A list of all families, together with their unique identifiers, can be found in Additional file 1, and a fasta file of our TE family library is provided as Additional file 2.

In a second step, we used these 1,819 families in a homology search with RepeatMasker [45] to identify all individual family members with a length of at least 100 bp in both genomes. We refer to these as TE copies but note that many of them are short TE fragments. We found a total of 92,798 TE copies. *A. lyrata *harbored most (69,942) copies, which comprised a total of approximately 25.1% of its genome. *A. thaliana *contained merely 22,856 copies that comprised approximately 15% of its genome. The below analysis is based on these copy numbers. A section on additional results (Additional file 3) contains a more conservative analysis that is based on elements greater than 2000 bp in length and that leads to the same conclusions.

Table 1 shows the copy numbers for the different TE classes in both genomes. It may seem unsurprising that *A. lyrata *contains more copies, because it has a larger genome. However, this higher abundance also persists if we take into account the different genome sizes. Specifically, the genome of the outcrossing *A*. *lyrata *contains 338 TE copies per million base pairs (Mbp), whereas *A. thaliana *contains only 192 copies per Mbp. The average length of one copy is approximately 753 bp, and is almost identical for elements in both species.

**Table 1:** Copy number distribution of the different TE classes in *A. thaliana *and *A. lyrata*

		*A. thaliana*			*A. lyrata*	
	
	Copy number	TE copies per Mbp	Percent of genome sequence	Copy number	TE copies per Mbp	Percent of genome sequence
LTR	6,784	56.9	6.9	18,558	89.8	12.9
non-LTR	2,243	18.8	1.2	6,844	33.1	2.6
DNA	12,631	106.0	6.7	40,118	194.1	9.0
unknown	1,198	10.1	0.2	4,422	21.4	0.7
Total	22,856	200.6	15.0	69,942	343.4	25.2

Even though *A. lyrata *contains more than three times as many TE copies than *A. thaliana*, there is no difference between the distribution of copies among the TE classes. In both species, the majority of copies, around 57%, are DNA transposons, followed by LTR elements (27%), non-LTR elements (10%), and non-classifiable elements (6%). LTR and DNA elements both represent around 8.1 Mb (6.8%) of genomic DNA in *A. thaliana*. In contrast, in *A. lyrata *LTR elements comprise with 26.7 Mb (13%) substantially more genomic DNA than DNA elements (18.6 Mb, 9%).

Out of the 1,819 element families we considered, the majority (1,447 families) have copies in both genomes. Twenty-six families exist only in *A. thaliana*, and 345 families exist only in *A. lyrata*. We were not able to identify any copies for one Repbase Update family (RU191, *Ta12*). Overall, our data show a more than tenfold excess of unique families in the outcrossing species *A. lyrata*. A list with the copy numbers of all our canonical elements can be found in Additional file 1.

### LTR elements

**Figure 1:**
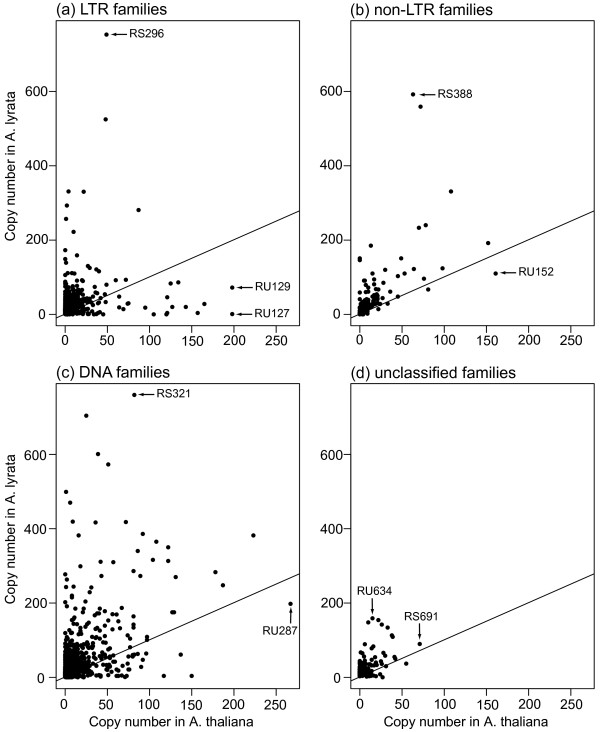
**Relationship between copy numbers in *A. thaliana *and *A. lyrata *for each family**. The panels show data for different TE classes, as indicated above each panel. The diagonal black line represents the line of equal copy numbers in both species. Families with a higher copy number in *A*. *thaliana *and *A. lyrata *thus correspond to points below and above the diagonal line, respectively. Families discussed in the text are highlighted and labeled by black arrows.

In most plant genomes, LTR elements are the most abundant elements. They are often responsible for a substantial increase in genome size [46-48]. In *A. thaliana*, the Repbase Update families *Athila3 *(RU127) and *Athila4a *(RU129) are the most abundant LTR families. They both have 198 copies. In contrast, in *A*. *lyrata*, the two families have only one and 72 copies, respectively, whereas the LTR family with the highest copy number in *A. lyrata *is RS296, one of our newly identified families. *A. lyrata *harbors 753 copies and *A*. *thaliana *49 copies of this family. RS296 is also the family with the highest overall copy number. Out of the 678 LTR families we studied, 131 families are unique to *A. lyrata*, and 11 families are unique to *A. thaliana*. Figure 1A shows a scatter plot of the copy numbers of LTR elements in *A. thaliana *and *A. lyrata*. The figure demonstrates that most families have fewer than 50 copies in *A. thaliana *and fewer than 150 copies in *A. lyrata*. It also shows that most families (84%, 570 of 678 families) have a higher copy number in *A*. *lyrata *(points above the diagonal line). It is also noted that the families with many copies in one species often do not have many copies in the other species. The three most abundant families in both species are highlighted.

A previous experimental study compared copy numbers for one LTR family between *A. thaliana *and *A*. *lyrata*. It found a slightly higher average copy number in *A. thaliana *(17.45 copies) than in *A. lyrata *(15.88 copies) [19]. This family also has higher abundance in *A. thaliana *in our data (see Additional file 3 for details), but our data also show that only a minority of families have this property.

### Non-LTR elements

Non-LTR elements contribute the least to TE abundance in both species. We identified only 143 non-LTR families. Only two of the non-LTR families are unique to *A. thaliana*, whereas 15 families are unique to *A*. *lyrata*. Non-LTR elements can be further subdivided into long and short interspersed nuclear elements (LINEs and SINEs). In *A. thaliana*, the non-LTR family with the highest copy number is the LINE *AtLine1a *(RU152) which contains 161 copies in *A. thaliana*, and 110 copies in *A. lyrata*. In *A. lyrata*, the non-LTR family with the highest copy number is also a LINE (RS388), with 592 copies in *A. lyrata*, but only 63 copies in *A. thaliana*. This family is also the non-LTR family with the highest overall copy number. The scatter plot in Figure 1B shows that, as for LTR element families, most non-LTR element families have more copies in *A. lyrata *(90%, 128/143 families). The majority of families have fewer than 50 copies in *A*. *thaliana *and fewer than 100 copies in *A. lyrata*.

A previous study based on more limited data compared the abundance of non-LTR families between the two species [19], and found that both families have higher copy numbers in *A. thaliana*. Our observations agree partly with these findings (see Additional file 3 for details).

### DNA elements

DNA elements have the highest copy number in both the *A. thaliana *and the *A. lyrata *genome. The most abundant DNA element families in these genomes are *Atrep3 *(RU287) with 267 copies and the RS321 family with 760 copies, respectively. Both families (*Atrep3 *(RU287) and RS321) belong to the DNA superfamily of Helitrons. This DNA superfamily was first identified almost 10 years ago in *A. thaliana *and *C. elegans *[49]. Helitrons are likely to use a rolling circle replication mechanism that allows them to capture host gene fragments [50]. Helitrons, also called *Basho *elements in *A. thaliana*, contribute around 2% of the *A. thaliana *genome, or one-fifth of the total TE DNA [41,51]. In *A. lyrata*, only a few Helitron families have been identified so far [50]. Our approach identified a large number of new Helitron elements in both *A. lyrata *and *A. thaliana*. In both species Helitrons make up more than 11% of TE copies.

In general, DNA elements are represented by 822 families, of which 12 families are unique to *A. thaliana *and 150 families are unique to *A. lyrata*. Figure 1C shows that most families have a higher abundance in *A*. *lyrata *than in *A. thaliana*, as we already observed for the other major element classes. Most families have fewer than 50 copies in *A. thaliana *and fewer than 100 copies in *A. lyrata*.

In a first comparison of TE abundance between the predominantly selfing *A. thaliana *and the outcrossing *A. lyrata*, Wright *et al*. [18] studied the *Ac*-like III transposon family in both species and found slightly more elements in *A. thaliana *[18]. Lockton and Gaut [19], however, later repeated this analysis and found on average more *Ac*-III copies in *A. lyrata *(22 copies) than in *A. thaliana *(12.5 copies). The *Ac*-like III family belongs to the widespread *hAT *superfamily which is responsible for various morphological changes [52] and chromosomal mutations [53]. Many *Ac*-like families in *Arabidopsis *seem to have transposed in recent evolutionary history [41,54]. Our analysis supports the results of Lockton and Gaut. We find a total of 26 copies in *A. thaliana *for this family, and a more than fivefold higher number in *A*. *lyrata *(143 copies). The copies are quite short and range between 100 and 661 bp in length, with the canonical sequence being 594 bp long. The copies present in *A. thaliana *are on average (276 bp) longer than the copies in *A. lyrata *(193 bp on average).

### Unclassified elements

We were not able to classify 176 (9.7%) of families identified during our RepeatScout search. These unclassified families show distributions similar to those of our classified families. Specifically, most families have a higher copy number in *A. lyrata*, as can be seen in Figure 1D. One family is unique to *A. thaliana *and 49 families are unique to *A. lyrata*. The family with the highest copy number in *A. thaliana *(71 copies) is RS691, the family with the highest copy number in *A. lyrata *(159 copies) is RS634.

### A systematically higher abundance of TEs in *A. lyrata*

**Figure 2:**
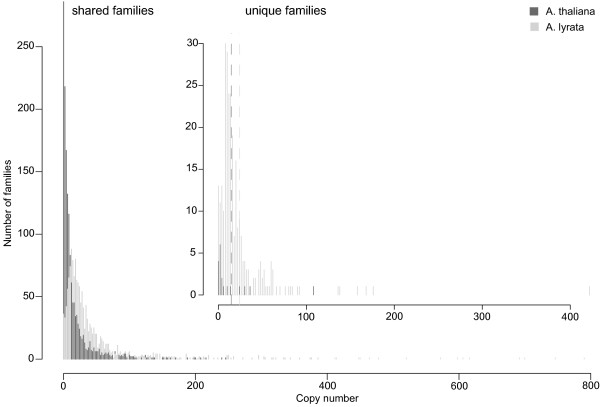
**Copy number distribution of TEs in both genomes**. The figure shows a histogram of element copy number (horizontal axis) divided into families shared between the two genomes and unique families (inset). Dark grey bars represent the number of families in *A. thaliana *and light grey bars in *A*. *lyrata*. The dashed lines of the same shading indicate the average copy number per family for the respective species. We note that for each copy number value on the horizontal axis, the bars for both *A*. *thaliana *and *A. lyrata *originate at a value of zero on the vertical axis. Thus, bars are not stacked, despite their visual appearance, and do not represent the sum of copies in both species. We used this format to ensure visual clarity despite the large number of bars in each histogram. Notice the different vertical scales for shared and unique families.

Next we compared the copy numbers of those 1,447 TE families with copies in both genomes. Only 20 of these families have equal TE copy numbers in both genomes; 200 families have a higher copy number in *A*. *thaliana*, and the vast majority of families (1,227 families) have a higher copy number in *A. lyrata*. The higher copy numbers are evident from Figure 1, because the majority of points are present above the diagonal line that indicates equal copy numbers in both genomes (see also Figure S1 in Additional file 3 for a representation on a logarithmic scale). This also holds if we take differences in genome size into account (Figure S2 in Additional file 3). The number of TE copies per family also differs systematically. Here, the *A. lyrata *genome contains an average of 47 copies per family, compared to only 17 copies per family in *A. thaliana*. In addition, the maximal copy number is much higher in *A. lyrata *(760 copies) than in *A. thaliana *(267 copies). If we compare the overall copy number distributions between families present in both species, as shown in Figure 2, we find a significant increase in copy number in *A. lyrata *(*P <*10^-15^, Wilcoxon rank sum test). In sum, the predominantly selfing *A. thaliana *has systematically fewer TE elements.

The same patterns hold for the TE families unique to *A. thaliana *and *A. lyrata*, as shown in Figure 2 (*P *= 5.3 × 10^-4^, Wilcoxon rank sum test). First, the number of TE families unique to *A. lyrata *(345 families) exceeds by more than tenfold the number of families unique to *A. thaliana *(26 families). Second, the average number of family members is higher in *A. lyrata *(20 members) than in *A. thaliana *(17 members). Finally, the maximum number of 277 copies of any one family is much higher in *A. lyrata *than in *A. thaliana*, where the largest family has only 120 copies.

We also asked whether similar patterns hold when we analyze our four TE classes LTR, non-LTR, DNA, and unclassified TEs, separately. The answer is yes. In each class, we find a higher average copy number in *A. lyrata*. The difference in copy number is significant for shared families of all classes (*P <*10^-11 ^for each class), and for unique families of LTR and DNA transposons (*P *= 0.01 and *P *= 0.001, respectively, Wilcoxon rank sum test). In addition, the number of unique families and the maximal copy number is always higher in *A. lyrata *than in *A. thaliana*. The only exceptions are the average copy numbers for unique LTR and unclassified families. Here, we find a higher average copy number in *A. thaliana *(29.9 and 27 copies for LTR and unclassified elements, respectively) than in *A. lyrata *(20.0 and 15.2 copies for LTR and unclassified elements, respectively). A full report for all classes can be found in Table S1 in Additional file 3. In addition, the higher TE copy number in *A. lyrata *also persists if we normalize for genome size (Figure S2 in Additional file 3). For all TE classes taken together, more than 74% of all families have a higher copy number per Mbp in *A. lyrata*.

The increase in copy number in *A. lyrata *might have occurred through an increase in copy number for elements in a few subfamilies. (We define a subfamily as a set of highly similar sequences within one family.) Alternatively, it might have occurred through an increase in copy number for most subfamilies. To distinguish these two scenarios, we constructed unrooted phylogenetic trees based on the multiple alignments of TE families. If only a few subfamilies had expanded in size, we would expect to find phylogenetic trees where most subfamilies have similar size in both species, and where only a few subfamilies have expanded dramatically. In contrast, if most subfamilies expanded in size, we would expect most subfamilies to have a higher copy number in *A. lyrata*. We excluded all sequences smaller than 200 bp from this analysis, because they are too short for phylogenetic reconstruction. Figure S3 in Additional file 3 shows examples of unrooted phylogenetic trees for two representatives from each of the major element classes (LTR, non-LTR, and DNA elements), with red branches indicating copies in *A. thaliana *and blue branches indicating copies in *A. lyrata*. In general, TE elements show very different phylogenetic relationships in the two *Arabidopsis *species. For most families, different subfamilies are likely to have been present before the split of the two species (e.g., Figure S3A,C in Additional file 3), and each single subfamily expanded differently in *A. thaliana *and *A. lyrata*. For some families, however, a division into subfamilies occurred after the split (e.g., Figure S3F in Additional file 3). Overall, our phylogenetic analysis suggests that the increase of element copy number in *A. lyrata *is caused by a broad range of subfamilies and not just by a few subfamilies. A more detailed discussion of the phylogenetic trees can be found in Additional file 3.

### TE insertions are more recent in *A. lyrata*

**Figure 3:**
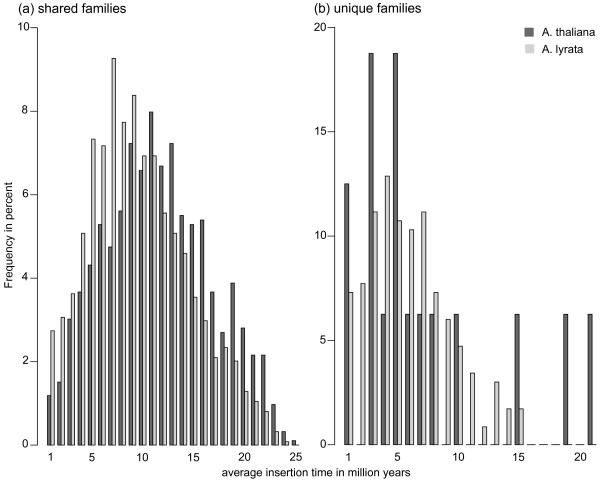
**Average insertion time distribution for each family**. The average insertion time was calculated based on the average nucleotide divergence between all copies of one family. **(a) **Histogram for shared families; **(b) **histogram for unique families. The percentage of families with a given age are represented by dark grey bars for *A. thaliana *and light grey bars for *A. lyrata*. The mean average insertion time for shared families is 11.1 Mya for *A. thaliana *and 9 Mya for *A. lyrata*, respectively. For unique families it is 6.7 and 5.5 Mya, respectively. The mean of the average insertion time for shared families is significantly smaller in *A. lyrata *(*P <*10^-15^, Wilcoxon rank sum test). Notice the different scales for shared and unique families.

If recombinational spreading is important for TE dynamics, as suggested by the lower TE copy numbers in *A. thaliana*, we expect to find that TE element insertions are on average older in the selfing species. The reason is that once a species becomes selfing, fewer insertions would go to high frequency or fixation, and thus become detectable by our approach. To find out whether this is the case, we created multiple sequence alignments for all elements in each of our 1,819 families. From these alignments, we estimated the average insertion time of elements in each family (see 'Methods' section for details). Overall, we find that TEs in *A. lyrata *have more recent average insertion times (8.5 ± 0.3 Mya) than TEs in *A. thaliana *(11.0 ± 0.4 Mya). This holds for families present in both genomes (11.1 ± 0.3 Mya versus 9.1 ± 0.3 Mya; see Figure 3A, for *A. thaliana *and *A. lyrata*, respectively), and for unique families (6.7 ± 3.1 Mya, 5.5 ± 0.4 Mya, for *A. thaliana *and *A. lyrata*, respectively, see Figure 3B). However, only the average time of insertions for shared families is significantly different among the two species (*P <*10^-15^, Wilcoxon rank sum test). We note in passing that shared families show on average an earlier insertion time than unique families (Figure 3), as one would expect if some unique families originated after the two species split. Within one family, insertion times can range from less than 100,000 years ago to more than 28 Mya. For example, more than 66% of TE families (1,171 families) have elements that inserted fewer than 1 Mya in *A*. *lyrata*. In contrast, fewer than 16% of families (240 families) have inserted that recently in *A. thaliana*.

### Number of insertions decreased recently in *A. thaliana*

Recent studies suggest that predominant selfing in *A. thaliana *arose 0 to 413,000 years ago, assuming 5 Mya as the approximate splitting time from *A. lyrata *[36,39]. Because the mating system may influence the evolutionary fate of TEs, we wished to estimate the insertion time for each element, to find out whether a change in successful insertions may have occurred in *A. thaliana *around that time. Our observations in the previous section, namely the lower abundance of recent insertions, and the higher average age of TE copies in *A. thaliana*, already hint at this possibility. We estimated the divergence time between two closest TEs as a proxy of insertion time. We note that we refer for brevity to insertion events throughout, but strictly speaking we can only detect insertion events for which both original and new copies exist in the studied genome sequences. These are preferentially insertions that have appreciable population frequency or that are fixed, and that can thus be found in the studied strains. Many more insertion events may have occurred but were lost from the genome's evolutionary record or they may be found in other individuals of the species. Thus, our estimate provides an upper limit of the age of the TEs. We note that for the same reason, successful insertion events cannot provide any information about insertion rates. For example, the insertion rate might be higher in *A. thaliana *than in *A. lyrata*, but a higher fraction of TE insertions might also get lost from the genome in *A. thaliana *before they become established in the population.

**Figure 4:**
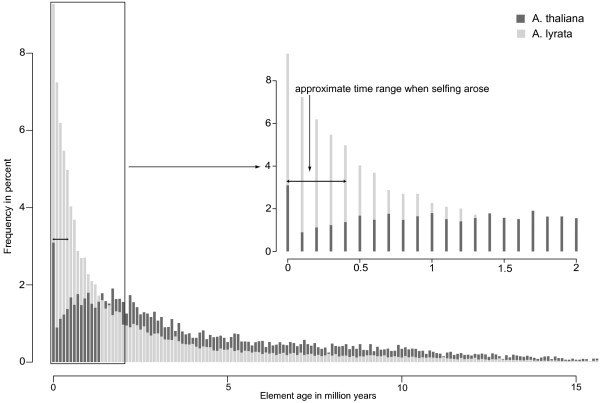
**Insertion time distribution for all elements**. A histogram of the number of elements with a given insertion time (horizontal axis). Values on the vertical axis are given as percent of the total number of elements. Elements in *A. thaliana *are represented by dark grey bars, and elements in *A. lyrata *by light grey bars. For visual clarity, the figure only shows elements with an insertion time less than 15 Mya. Only few elements were inserted even earlier. The inset shows the frequency of elements younger than 2 million years. The double-headed arrow indicates the approximate time range when selfing arose in *A*. *thaliana *[36,39]. We note that for each copy number value on the horizontal axis, the bars for both *A. thaliana *and *A. lyrata *originate at a value of zero on the vertical axis. Thus, bars are not stacked, despite their visual appearance, and do not represent the sum of copies in both species. We used this format to ensure visual clarity despite the large number of bars in each histogram.

Once a TE has inserted into a specific site in a genome, it is subject to excision and other mutation events that may inactivate it and eventually eliminate it from the genome [55]. The likelihood that any one element experiences such a mutation increases with the age of the element, that is, with the residence time of the element at that site. Thus, one would expect that any one element found at a specific site in a genome is more likely to be recent than ancient, because recently inserted elements are less likely to have suffered such mutations. Moreover, if the probability that any one mutation occurs is constant per unit time and independent of previous events, one would expect a roughly exponential distribution of element age. The age distribution of *A. lyrata *TEs adheres to this expectation (Figure 4, light grey bars). It corresponds roughly to an exponential distribution with a half life of 615,400 years (Figure S4B in Additional file 3). The number of elements younger than 2 million years fits the exponential distribution especially well. Older elements are slightly overrepresented, indicating a slightly elevated survival time for such elements, possibly due to smaller deleterious effects that their insertions may have, or perhaps even due to advantages they may provide for the host. In contrast, the age distribution of TE copies in *A*. *thaliana *is markedly different (Figure 4, dark grey bars). First, the mean element age is significantly higher in *A. thaliana *(*P <*10^-15^, Wilcoxon rank sum test). Second, the decline in element age in *A. thaliana *is not as rapid as for *A. lyrata*. Third, a small drop in abundance occurs at an approximate element age of 0.5 to 1.6 Mya (Figure 4 and inset). Fourth and most important, a more pronounced drop in element abundance also occurs during the last 10% of the time interval since the split between *A. thaliana *and *A*. *lyrata*, corresponding to elements less than 500,000 years old. This time interval is very similar to the estimated time interval during which self-incompatibility was lost in *A. thaliana *(0 to 413,000 years ago) [36,39] (black double-headed arrow in Figure 4). As we discussed earlier, our estimates are upper boundaries of insertion times, and thus the elements we study might have inserted more recently. Even so, the drop of element abundance we observe would fall into the time range of 0 to 413,000 years ago when selfing arose. If we use the minimum and maximum substitution rate for *A. thaliana *instead of the average substitution rate as given in [31], the time intervals for the drop in element abundance become 0.2 to 0.3 and 0.2 to 0.8 Mya, respectively (Figures S5,S6 in Additional file 3). The drop in copy number during the time predominant selfing arose in *A. thaliana *is consistent with a decrease in the rate of successful insertions, because of a lack of recombinational spreading caused by selfing (see 'Discussion' section).

Similar age distributions exist when we consider LTR, non-LTR, DNA, and unclassified TEs separately (Figures S7, S8, S9, S10, respectively, in Additional file 3). A rapid decrease of element number with increasing age occurs in *A. lyrata*. The mean element age is always significantly shorter for *A. lyrata *(*P <*10^-15^, Wilcoxon rank sum test). For LTR and DNA elements, the classes with the highest copy numbers, we observe a drop in element number for elements younger than 0.5 and 0.4 Mya.

**Figure 5:**
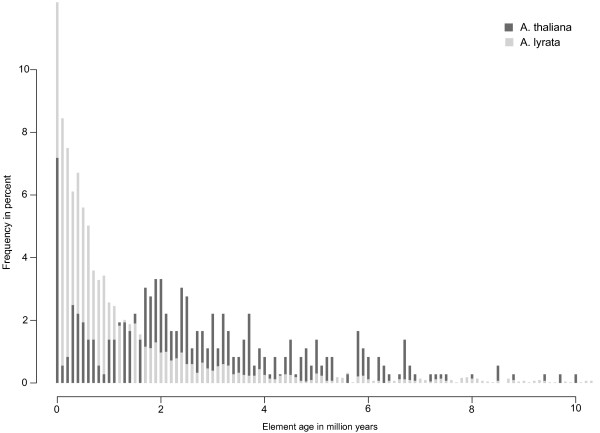
**Insertion time distribution for elements belonging to families unique to one genome**. Analogous to Figure 4, but only for families unique to one of the two genomes. The figure shows a histogram of the number of elements with a given insertion time (horizontal axis). Values on the vertical axis are given as percent of the total number of elements. Elements unique to *A. thaliana *are represented by dark grey bars, and elements unique to *A. lyrata *by light grey bars. For *A. thaliana *only 363 elements belong to families unique to this species. We note that for each copy number value on the horizontal axis, the bars for both *A. thaliana *and *A. lyrata *originate at a value of zero on the vertical axis. Thus, bars are not stacked, despite their visual appearance, and do not represent the sum of copies in both species. We used this format to ensure visual clarity despite the large number of bars.

While TE families present in both genomes were already present before the split of the two species, some families unique to one of the genomes might represent families which arose after the split from the common ancestor. The insertion time distribution of these elements might thus provide a further indication of how the evolutionary dynamics change after a change in mating system. Figure 5 shows the element age distribution for unique families in *A. thaliana *(dark grey bars) and *A. lyrata *(light grey bars). It is approximately exponential again for *A. lyrata*, with a halflife of approximately 540,000 years (Figure S4D in Additional file 3). There are too few unique elements in *A. thaliana *to ascertain the shape of their age distribution with confidence (Figure S4C in Additional file 3).

### Results remain unchanged for a more conservative TE library

A disadvantage of identifying TEs *de novo *is that any algorithm might return several sequence fragments instead of one full-length sequence for a highly diverged member of a TE family. We were concerned that such sequence fragmentation might influence our analysis. We therefore created a second element library containing only RepeatScout sequences with a length of at least 2000 bp and all Repbase Update sequences. We repeated our analysis with this conservative set to validate our results. This analysis leads to the same conclusions as the analyses we reported above (see Additional file 3).

## Discussion

The influence of the mating system on TE dynamics has attracted considerable interest [12-14,17,19,21]. The availability of the genome sequences of the predominant selfing plant species *A. thaliana *(strain Col-0) and its close outcrossing relative *A. lyrata *(strain MN47) allowed us to provide pertinent genome-scale evidence from two closely related species with different mating systems. Most TE families and subfamilies were already present before the split of the two species from their common ancestor, yet they show different abundances and dynamics in both species. We find, first, a significantly lower number of TE copies in the predominantly selfing *A. thaliana *than in the outcrossing *A. lyrata*. Second, TEs present in *A. thaliana *are, on average, older than TEs present in *A. lyrata*. Third, we observe a pronounced decrease in new insertions in *A. thaliana *that occurred long after the speciation with *A. lyrata *(0.5 to 1.6 Mya), assuming a speciation time of 5 Mya. Fourth, we see a pronounced decrease of insertions younger than 0.4 to 0.5 Mya in *A. thaliana*, the approximate time when this species became predominantly selfing.

### Higher TE copy numbers in *A. lyrata*

We identified more than 22,000 TE copies in *A. thaliana *(15% of genomic DNA) and more than 69,000 copies in *A. lyrata *(25% of genomic DNA). Fifteen percent of genomic DNA are derived from TEs in *A*. *thaliana*, which exceeds by one half the amount previously reported from the genome sequence (10%) [22]. This increase in estimated genomic TE content is a result of our search method, which not only uses known TEs as query sequences but also involved a *de novo *search (see 'Methods' section). Our findings agree with two other very recent studies reporting a genome wide TE content for *A. lyrata *[23,42]. One study identified 22,818 TE copies in *A. thaliana *and 67,033 in *A. lyrata*, very close to the numbers we identified. This study focused on the effect of small interfering RNAs directed against TEs on gene expression, whereas we here focused on the comparative abundance distribution and insertion ages of TEs [42]. The other study [23] also identified more TE copies in *A. lyrata *than in *A. thaliana*, but the overall copy numbers it identified (80,225 and 26,990 copies, respectively) were somewhat higher than ours. This higher estimated copy number is caused by details of the search procedure, because both previous studies use an approach very similar to ours. Specifically, they first identified elements *de novo *and then used these elements as a search library in a RepeatMasker search. In fact, both studies use the same library for their homology search (the two papers share some authors), which means that they must use different parameters in the RepeatMasker search. Unfortunately, they did not publish the parameters they used in their RepeatMasker searches. Relatedly, we note that Hollister *et al*. [42] concatenated candidate TEs of the same family into one TE copy if they were located less than 100 bps from one another [42], which results in a lower copy number in the study by Hollister *et al*. [42] compared to Hu *et al*. [23]. In contrast, we only allowed TE copies with a length of at least 100 bps in our RepeatMasker search. Hu *et al*. [23] do not mention if they used any constrains in their homology search. It may be the case that these authors also counted shorter hits in the homology search as TE copies, which could explain the higher number of TE copies in both species.

The identification of new TE elements, as opposed to reliance on already known elements, can influence TE abundance estimates dramatically. The Helitron superfamily of DNA elements provides an example. All previously known 36 Helitron families for *A. thaliana *from Repbase Update have copies in both genomes. These copies contribute to a total length of 1.8 and 1.2 Mb Helitron-derived DNA in *A. thaliana *and *A*. *lyrata*. Based on this observation, Helitrons appear more abundant in *A. thaliana*. Our *de novo *search identified 71 new Helitron families. A homology search with these new families not only identified 15 of these 71 families as unique to *A. lyrata*, it also increased the total amount of Helitron derived DNA to approximately 2 Mb in *A. thaliana *and to approximately 4.6 Mb in *A. lyrata*. As a result, the genome of *A*. *lyrata *contains more than twice as much Helitron-based DNA as *A. thaliana*.

### Arabidopsis genomes contain diverse representatives of all known TE superfamilies

*A. thaliana *and *A. lyrata *both contain diverse representatives of all known superfamilies of TEs, with DNA transposons having the most copies in both genomes (approximately 57% of TE copies in both genomes). In *A. thaliana*, the total amount of genomic DNA covered by LTR and DNA elements is very similar approximately 8.1 Mbp (6.8% of genomic DNA). In contrast, in *A. lyrata *this amount is much greater for LTR elements (26.7 Mbp, 13%) than for DNA elements (18.6 Mbp, 9%). The higher amount of LTR-based genomic DNA in *A. lyrata *agrees with data from all other flowering plants studied so far, where LTR retrotransposons contribute most to a genome's TE content. In some genomes, like maize, wheat, and barley, LTR elements contribute more than 60 to 80% of genomic DNA [56], and these LTR elements play a pivotal role in rapidly increasing genome sizes in these organisms [57].

### Comparison to other previous results

Before the availability of the *A. lyrata *genome sequence, only a few studies compared TE abundance between *A. thaliana *and *A. lyrata *[18,19]. These studies focused on only a few families. Because no TE families had been identified for *A. lyrata*, these studies were based on families from *A. thaliana*. The use of *A. thaliana *sequences and the lack of information about families unique to *A. lyrata *may lead to underestimates of element numbers in both species, but especially in *A. lyrata*. Because our search method was able to identify new families in *A. lyrata*, and also identify families specific to *A. lyrata*, our results might differ from previous observations. This is indeed the case, as discussed in greater detail in Additional file 2 and shown in Table S2 in Additional file 3. In most cases, we identified more TE copies than previous studies, especially in *A. lyrata*, although the individuals used for our analysis were not the same as those used in previous analyses (and different individuals may have different copy numbers). For example, for *Ac*-like elements, Lockton and Gaut identified 12.5 and 22 copies, compared to 26 and 143 copies identified by us in *A. thaliana *and *A. lyrata*, respectively [19].

### Reduction of unique families in *A. thaliana *and higher copy numbers in *A. lyrata*

Most of our 1,819 TE families are present in both *Arabidopsis *genomes. Only 371 families are unique to one species. These species-specific families may have evolved from a related family after the split. Alternatively, they may have been present in the common ancestor, but were lost in one of the species, or they may have diverged beyond recognition. The latter possibility seems unlikely, given the recent divergence of the two species.

Relatedly, the average insertion time of unique families (Figure 3) indicates that such families are on average younger than shared families. What is more, unique families have an average insertion time that is more recent than the split between *A. thaliana *and *A. lyrata*. This observation suggests that new families have indeed evolved since the two species split.

It is also remarkable that the 371 unique families are not equally distributed between both species. The majority of 345 families is unique to *A. lyrata*, and only 26 families are unique to *A. thaliana*, which is more than a tenfold difference in family number. In addition, the families unique to *A. lyrata *have a higher average copy number than the families unique to *A. thaliana*.

Totally 1,447 TE families are present in both genomes, but at substantially different copy numbers. Specifically, *A. lyrata *contains on average almost three times as many copies for each shared TE family as *A. thaliana*. Shared TE families are families that have been present in the common ancestor of the two species. Not only is this the case for most TE families, most of these families were already divided into several subfamilies, as shown by phylogenetic trees of several families (Figure S3 in Additional file 3). We do not find a general pattern for subfamily evolution. Some subfamilies experienced recent insertion events in one or both species, while other subfamilies are represented only by a single copy. However, there are many more recent successful insertions in *A. lyrata*. For the minority of families whose subdivision occurred after the split between the two species, a clear separation between elements in *A. thaliana *and *A*. *lyrata *can be seen, as exemplified by the family tree in Figure S3F in Additional file 3.

Our observations are consistent with the findings by Zhang and Wessler [58] who showed that almost all TE lineages are shared between *A. thaliana *and *Brassica oleracea *[58], two species that diverged from their common ancestor approximately 15 to 20 Mya [59].

### The change from outcrossing to selfing may have affected TE insertion in *A. thaliana*

The evolution of self-fertilization by the loss of self-incompatibility has been regarded as one of the most prevalent evolutionary trends in plants [60,61]. It is often accompanied by changes in chromosome numbers, in the abundance of TEs, in intron sizes, and in morphological traits such as flower size [9]. Recently, the origin of self-compatibility has been studied using various kinds of data [37,62]. Molecular genetic, evolutionary genetic, and evolutionary genomic studies in *A. thaliana *[36,38], as well as phylogenetic studies of the family Solanaceae [63] have shown that self-compatible species are short-lived.

Population genetic studies of many Brassicaceae species suggested that self-compatibility originated during the most recent glacial cycle [25,36,64-68]. Specifically, it originated 20,000 to 50,000 years ago in *Capsella rubella*, as well as about 150,000 and 12,000 to 48,000 year ago in two lineages of *Leavenworthia alabamica*, and 0 to 413,000 years ago in *A. thaliana*, assuming the substitution rate estimated by Koch *et al*. [31]. The focus of previous studies was limited to the analysis of the self-incompatibility locus or other protein coding genes. Our study of the age distribution of genome-wide TEs provides a unique dataset relevant to the evolution of self-compatibility.

To find out whether the lower copy number in *A. thaliana *could be explained by a change in the mating system, we asked whether we can observe a difference in the number of new TEs after selfing arose in *A*. *thaliana *approximately 0 to 413,000 years ago [36,39]. To this end, we estimated the insertion time distribution of all TE copies in both species. This distribution differs dramatically between the species. *A*. *lyrata *elements show an approximately exponential age distribution with a vast majority of recently inserted elements, and a rapid decrease of element number with element age. In contrast, the TE age distribution of *A. thaliana *is markedly non-exponential, and differs in several other respects, including a drop in element number for elements younger than 0.5 million years (Figure 4). The decrease of elements younger than 0.5 million years is consistent with the estimated time interval for the origin of predominant selfing in *A. thaliana*.

For an anciently selfing species, one would expect the TE age distribution to be exponential, just as in an outcrossing species. However, this distribution should be shifted toward a lower number of recent insertions compared to an outcrossing species, if selfing reduces the successful propagation of TEs, for example through a lack of recombinational spreading. *A. thaliana *is not an anciently but a recently selfing species, and this simple scenario may thus not apply to it. Rather, the age distribution of its TEs might be a superposition of two exponential distributions, one each for elements that inserted before and after the recent change in mating system. The age distribution of elements unique to *A. thaliana *might be informative about the second of these two distributions, if such families originated since the separation of the two species. Unfortunately, this distribution contains too few (363) elements to ascertain with confidence whether it is exponential (Figure S4C in Additional file 3). However, we note that the number of the most recent insertions is much smaller in *A. thaliana *than in *A. lyrata *(26 versus 525 insertions). Although a superposition of two distributions might explain the lower number of insertions before selfing arose in *A. thaliana*, other factors might also influence TE dynamics in *A. thaliana*. Many outcrossing species first become partially selfing, and later predominantly selfing. If this is the case in *A. thaliana *it might explain why we see fewer elements between the time selfing arose and the time when the two species split. Unfortunately, not much is known about the life history and breeding system of the ancestors of *A*. *thaliana *[37,39,40].

Because we used the substitution rate from *A. thaliana *to estimate the element insertion times in both genomes, the question arises how our observations would be affected if the substitution rate in *A. lyrata *is actually much lower than in *A. thaliana*. Could the larger number of young elements in *A. lyrata *be explained by this difference? In this regard, we note that a lower substitution rate in *A. lyrata *would only affect the insertion time distribution in *A. lyrata *(the decrease in its element numbers with age would be less rapid) but it would not change the insertion time distribution in *A. thaliana*.

Overall, the abundance of TEs in both our study species, the age distribution of these TEs, and the change in this distribution at about the time when selfing arose point to an important role for selfing in determining the fate of TEs in a genome. Specifically, they suggest that insertions which are successful and spread through a population are rarer in selfing species. A prominent candidate cause is the lack of recombinational spreading that TEs may experience in selfing species [16,17]. However, we note that our data cannot exclude that other causes, for example ectopic recombination, may contribute to the differences we observe.

### Caveats

No comparative analysis like ours can *prove *that a specific cause, such as outcrossing or selfing, is solely responsible for differences in TE abundance and evolutionary dynamics. Factors other than selfing may contribute as well. Among them are differences in genome size. Selection can favor small genomes, for example during the evolution of an annual life cycle [7-9,23]. (It is unknown when *A. thaliana *became an annual [10].) In addition to having a smaller genome than *A. lyrata*, *A. thaliana *also has fewer chromosomes. Like many other chromosomal rearrangements, chromosome fusions could result in the loss of many TEs around centromeric regions. Based on these observations, one might argue that the differences in TE abundance between the two species could be caused solely by differences in genome size. Our observations suggest otherwise. Even if we control for genome size differences, TEs are more abundant per Mbp of genomic DNA in the outcrossing species (Table 1 and Figure S2 in Additional file 3). Genome size differences are thus probably not a major factor confounding the results of our analysis.

A second potential confounding factor is effective population size. In small populations, selection against weakly deleterious TE insertions is less effective, and TEs could thus accumulate in a genome [8]. However, in this regard we note that a recent analysis based on polymorphisms in many genes yielded very similar estimates of effective population sizes for *A. thaliana *(1.27 × 10^5^) and *A. lyrata *(1.38 × 10^5^) [69]. Thus, effective population size differences are not likely to account for the different patterns of TE dynamics we see in *A. thaliana *and *A. lyrata*.

A third caveat regards the fact that we only have access to the genomes of one individual from a population of individuals for each of the two species. It is well known that TE insertions may be polymorphic in populations of the organisms we study [18]. One can therefore not assume that a TE insertion found in a single individual would be fixed, that is, that it would also exist in all other individuals of the population. Many or most TE insertions may even occur at low population frequencies. Using a sample of one individual from a population leads to an ascertainment bias which favors the discovery of TEs that occur at high frequency or that are fixed in a population. This bias has at least two consequences. First, it can lead us to underestimate the number of TEs that occur in a genome, because our sample will miss many TEs that have low population frequencies. Second, it can lead us to overestimate the average age of TEs, such that TEs are younger than they appear. The evolutionary dynamics of TEs in a population is complex, and depends on multiple factors. Unfortunately, the available data do not allow us to estimate the error in our estimates caused by this limitation. Importantly, however, if we have overestimated insertion times, the reduction of insertions we observe could have occurred more recently than 500,000 years ago, but it would still fall into the time interval during which predominant selfing arose in *A. thaliana *(0 to 413,000 years ago).

Fourth, our insertion time estimates are only based on elements that are still present in a genome. For ancient TE families that have been present in a genome for a long time, the oldest elements may have diverged beyond recognition, or they may have become lost from the genome. This means that we cannot estimate from a family's oldest elements when the family first arose. This limitation is the reason why we used average insertion times (instead of maximal insertion times) as a proxy to compare family ages. Because most TEs in our analysis are recently inserted, the very few old TEs are probably not an important confounding factor for this aspect of our analysis. We also note again that both of our genomes would suffer from these problems to a similar extent.

Fifth, variation in evolutionary rate among elements and gene conversion could affect our insertion time estimates. Here again, we note that both of our genomes would suffer from these problems to a similar extent, such that these factors are not likely to compromise our comparative observations substantially.

Sixth, other differences that may be unrelated to the rise of selfing, such as DNA methylation levels [70], could affect TE abundance. We cannot conclusively exclude such unknown confounding factors. However, the observation that the TE age distribution changes around the time selfing originated in *A. thaliana *points to a link with selfing that is consistent with the suggestions of past workers [12,13].

## Conclusions

We comprehensively analyzed TEs in the predominately selfing plant *A. thaliana *and its close outcrossing relative *A. lyrata*. We found a substantially smaller number of TE copies in *A. thaliana *compared to the outcrossing *A. lyrata*. TE families and elements are on average younger in *A. lyrata*, indicating more successful recent transpositions in this species. *A. thaliana *shows a decrease in elements younger than the approximate time when selfing became the predominant mode of reproduction in this species. Our observations are consistent with evolutionary dynamics that render TEs less abundant in selfing species and more abundant in outcrossing species, such as the dynamics postulated by the recombinational spreading hypothesis.

## Methods

### Element identification

We extracted a total of 357 canonical TE sequences, prototypic sequences that either represent consensus sequences or a sequence example for a TE family, for *A. thaliana *from Repbase Update [43], a database containing repetitive DNA elements in eukaryotes. This set was divided into 165 DNA transposon sequences, 151 LTR retrotransposon sequences, and 41 non-LTR retrotransposon sequences.

Because no canonical sequences are available for *A. lyrata *and there may be unidentified TE families present in *A. thaliana*, we also attempted to find putative TE elements *de novo*. To this end, we used an approach similar to [42]. More precisely, we first used RepeatScout [44] with default settings for a *de novo *search of repetitive DNA in both genome sequences. A previous test of different *de novo *algorithms had identified RepeatScout as best suited for such a search [71]. The main idea behind it is to identify small repetitive regions as seeds (l-mers) and extend them. RepeatScout returns a consensus sequence for each repeat family it identifies. Because the output of RepeatScout contains all kinds of repeats, including TEs, low-complexity repeats, tandem repeats, multicopy gene families and pseudogenes, and segmental duplications, we had to apply several filtering steps to exclude all hits except likely TEs (see Additional file 3 for details), as has been suggested by the authors of RepeatScout [44].

Next, we compared our *de novo *element set with the canonical elements from Repbase Update, and excluded all elements from our set that showed more than 80% similarity to an element from Repbase Update, to avoid redundancy in our data. We assigned all elements that remained in our dataset after this exclusion step to different TE classes by using the programs RepClass [72] and TeClass [73] (see Additional file 3 for details). Our final set of TE family consensus sequences in *A. thaliana *and *A. lyrata *thus contains all element sequences from Repbase Update and all non-redundant *de novo *elements identified by RepeatScout. We will refer to this dataset as our library of TE families, in which each family is represented with one member.

In our next step, we used this library in a homology search to detect all members of each TE family within both genomes. We chose RepeatMasker [45], the most commonly used tool for homology-based repeat detection for this identification. Because we used our own repeat library, we increased the cutoff threshold to 250 from a default value of 225, and performed the most sensitive search possible. We allowed RepeatMasker to search also for low complexity and simple repeats to avoid false positive matches in regions containing such repeats. If the search algorithm identified overlapping elements, we only considered the element with the best score in our further analysis. In addition, we used the RepeatMasker (option a) to determine all pairwise alignments between elements in our library and corresponding copies identified in the genomes. We only considered element copies with a length of at least 100 bp for our analysis.

The problem with *de novo *identification algorithms is that they often return several fragmented sequences for one family instead of one full-length sequence. The fragmentation of a family sequence might influence the results of our analysis. We therefore constructed a second, more conservative set of TE families with all Repbase Update elements, and only RepeatScout sequences with a length of at least 2000 bp. We call this family set the 'conservative' set. We used this set, in addition to our library of TE families, for all analyses. In the main text, we only discuss the results from our full library. The complete results for the conservative set can be found in Additional file 3.

### Multiple alignment construction and phylogenetic analysis

We constructed three multiple alignments for each TE family in our library based on the pairwise alignments generated during the RepeatMasker search. The first alignment contained all the copies of this family present in both genomes, the second alignment contained only the copies identified in *A. thaliana*, and the third alignment is based only on copies from *A. lyrata*.

Based on the first multiple alignment (all copies in both genomes), we computed phylogenetic trees for each family using PhyML_aLRT [74] a version of PhyML [75], which incorporates an approximate likelihood ratio test to estimate the statistical support of the tree topology. This approach is superior to a bootstrap calculation with respect to accuracy and power, and it is computationally much more efficient [74]. The method assigns to each branch a statistical significance ranging from 0 (least significant) to 1 (highly significant). In calculating the trees, we used the default options of PhyML_aLRT, i.e., the HKY nucleotide substitution matrix, the proportion of invariable sites set to zero, and only one category of substitution rate [75]. We chose the *χ*^2^-based parametric branch support for approximate likelihood ratio tests [74], and excluded all sequences shorter than 200 bp because they are too short for phylogenetic reconstruction.

### Insertion time estimation

We calculated the pairwise DNA sequence identity between copies from one element family using the multiple alignments of the family. To this end, we employed the dnadist program from the PHYLIP package (http://evolution.genetics.washington.edu/phylip.html), and restricted ourselves to sequence pairs that overlapped by more than 200 bp in the multiple sequence alignment.

We then estimated the insertion time of any one element in a family by identifying the family member that had the highest nucleotide identity with this element (i.e., the lowest divergence *K*). We used the expression *T *= *K*/2*r *where *T *is the time to most common ancestry, *K *is the sequence divergence, and *r *is the substitution rate, as described by Bowen and McDonald [76]. For the substitution rate, we used an estimate of 0.015 substitutions per site per million years [31]. We calculated the average insertion time of elements in one family from the average divergence between all copies of that family.

## Competing interests

The authors declare that they have no competing interests.

## Authors' contributions

NC carried out all computational analyses. NC, TT, KS, and AW designed the study and wrote the manuscript. All authors read and approved the final manuscript.

## Supplementary Material

Additional file 1 **Copy numbers of TE families**. For each of our TE families (Repbase Update and newly identified families) the copy number in both genomes is listed. Each family has a unique identifier. Families derived from Repbase Update have the identifier RUxxxx and our newly identified families have the identifier RSxxxx, where xxxx stands for a one to four digit long integer, e.g., RU287. The unique identifiers are also used as fasta identifier in Additional file 2. Click here for file

Additional file 2 **Sequence library**. Nucleotide sequence of all TE families in fasta format. Each record (family) has a unique fasta identifier. Elements derived from Repbase Update have the identifier RUxxx and our newly identified families have the identifier RSxxxx, where xxxx stands for a one to four digit long integer, e.g., RU287. The unique identifiers are also used for the copy numbers of each family in Additional file 1. Click here for file

Additional file 3 **Additional material, figures, and tables**. This file contains additional methods, results, figures (S1-S14), and tables (S1-S2). Click here for file
