# Supplementary material for: The predominantly selfing plant Arabidopsis thaliana experienced a recent reduction in transposable element abundance compared to its outcrossing relative Arabidopsis lyrata
Source: Mob DNA. 2012 Feb 7;3:2. doi: 10.1186/1759-8753-3-2 (PMC3292453; doi:10.1186/1759-8753-3-2)
Supplement: Additional file 3 — Additional material, figures, and tables. This file contains additional methods, results, figures (S1-S14), and tables (S1-S2). [file 1759-8753-3-2-S3.PDF]

Additional Methods

Element identification

Because the output of RepeatScout in our de novo search for elements contains all kinds of repeats, including transposable elements, low-complexity repeats, tandem repeats, multicopy gene families and pseudogenes, and segmental duplications, we had to apply several filtering steps to exclude all repeats different from transposable elements, as has been suggested by the authors of RepeatScout [1]. We filtered low-complexity and tandem repeats with the help of Nseg [2] and Tandem Repeat Finder [3], if more than 50 percent of a hits sequence was annotated as low-complexity or as a tandem repeat. Furthermore, we excluded all repeats shorter than 100 bp, because known transposable elements are longer than this threshold. To exclude multicopy gene families and pseudogenes, we compared the consensus sequence of our RepeatScout repeats against all known gene sequences for the respective species. For *A. thaliana* we used the file TAIR9_seq_20090619.fasta in the Arabidopsis Information Resource (ftp://ftp.arabidopsis.org/home/tair/Sequences/blast_datasets/TAIR9_blastsets/) in this analysis. Because this file also includes sequences of transposable elements, we excluded them before this step of our procedure. Furthermore, we excluded genes encoding "unknown proteins" as they might belong to a transposable element which has not been yet characterized. For *A. lyrata* we obtained and used the file http://genome.jgi-psf.org/Araly1/Araly1.download.ftp.html. We excluded all repeat elements that overlapped a sequence in these two data sets over more than 50 percent of their length. In a next filtering step we performed a RepeatMasker [4] search to obtain the copy number of each repeat sequence in each of the two genomes. We did not count copies of a repeat that overlapped another repeat over 80 percent or more of their total length as a separate copy. Furthermore, we excluded all repeats from our data set with fewer than 10 copies in a genome, as suggested in [1]. In addition to the suggested filtering steps, we used Phobo, a tandem repeat search tool [5], to filter out tandem repeats missed by the Tandem Repeat Finder. We excluded all repeats where more than 20 percent of the sequence corresponded to tandem repeats. As a last step, we compared our data set with the canonical elements from Repbase Update, and excluded all elements with more than 80 percent similarity to an element from Repbase Update to avoid redundancy.

We then assigned all the elements remaining after this last step to different TE classes by using the programs RepClass [6] and TeClass [7]. RepClass uses different approaches for this classification, including homology search and structural features to identify the class (Retrotransposon, DNA transposon), subclass (LTR/non-LTR, DNA/Helitron), and superfamily of each element. The program TeClass on the other hand uses a machine learning approach to classify different elements. Neither of the programs were able to classify all remaining elements. We merged the results from both programs according to the following rules: If both algorithms returned the same classification, we used that classification. If an element was only classified by one of the algorithms, this classification was taken. If the two algorithms returned different classifications we used the result from RepClass if this algorithm classified the element based on a homology search and from TeClass otherwise. We want to note that we were not able to classify all elements and we can not prove that these unclassified elements are true TEs. However, for completeness we included them in our analysis.

Our final library of transposable element families in *A. thaliana* and *A. lyrata* contains all element sequences from our de novo search, as just described, together with all elements from Repbase Update.

Additional Results

Detailed comparison to previous experimental studies

Previous experimental studies used the polymerase chain reaction (PCR) to identify TE copies in the genomes of both species. We used the primer sequences from these studies as diagnostic sequences to match the previously described families to our families based on sequence identity.

LTR elements Lockton and Gaut (2010) studied one member family of the LTR superfamily *gypsy* in *A. lyrata* [8], and comparatively between *A. thaliana* and *A. lyrata* [9]. These authors found a slightly higher average copy number in *A. thaliana* (17.45 copies) than in *A. lyrata* (15.9 copies) [9]. The *gypsy* family these authors studied corresponds to two of our TE families, (*Athila2* (RU126) and *Athila6c* (RU136)). While both families show a high copy number in *A. thaliana* (157 and 121 copies, respectively) we were only able to identify 4 copies per family in *A. lyrata*, which is even lower than previously reported (15.9 copies) [9]. As the *gypsy*-like family already corresponds to two of our families, it is possible that there exists an even greater number of related families. Indeed, we find five more families identified by RepeatScout that share a high sequence identify to the diagnostic sequences. These families have a higher copy number in *A. lyrata* (161 copies in total) than in *A. thaliana* (17 copies in total). The greater total copy number of all seven families in *A. thaliana* (295 copies) than in *A. lyrata* (169 copies) supports previous observations [9].

non-LTR elements Lockton and Gaut (2010) compared one family of LINE and one family of SINE elements between *A. thaliana* and *A. lyrata* [9]. These families correspond to our families *Atline1a* (RB152) and *Atsb2* (RB169), respectively. The LINE family is the family with the highest copy number in *A. thaliana*. For the SINE family, we find 152 copies in *A. thaliana* and 192 in *A. lyrata*. We find that one family has a higher copy number in *A. thaliana* and the other in *A. lyrata*. This partly agrees with previous results [9].

Phylogenetic relationships between TEs in both species

To examine relationships between copies in different genomes, we constructed unrooted phylogenetic trees based on the multiple alignments of TE families. We excluded all sequences shorter than 200 bp from this analysis, because they are too short for phylogenetic reconstruction. Additional Figure S3 shows examples of unrooted phylogenetic trees for two representatives from each of the major element classes (LTR, non-LTR and DNA elements), with red branches indicating copies in *A. thaliana* and blue branches copies in *A. lyrata*. These examples are representative of the phylogenetic relationships we see in our data set.

Panels a and b represent phylogenetic trees for two LTR element families, that is, RS296 and RU126. RS296 (panel a) is a family newly identified in this study. With a total of 802 copies, it has the fourth highest copy number in both genomes (49 copies in *A. thaliana* and 753 copies *A. lyrata*), and the highest copy number for any LTR family. Because most of the copies are present in *A. lyrata*, most of the branches are blue. Some of the *A. thaliana* copies are spread throughout the tree in different subclades. These copies usually are more divergent (have longer branches) than the *A. lyrata* copies present in the same subclade. The remaining *A. thaliana* copies cluster together and form their own subclade. In the second LTR tree (panel b), most of the RU126 (*Athila2*) copies are present

in *A. thaliana*; the five copies present in *A. lyrata* form two small subclades (157 and 5 copies in *A. thaliana* and *A. lyrata*, respectively.

The second row (panels c and d) shows the phylogenetic relationship between two non-LTR families. The first tree (c) represents the relationship between copies of the LINE family RS388, which is the most abundant non-LTR family in our set (655 copies in total). Copies from the two different species appear intermingled more than for the two LTR element trees in panels a and b, but species-specific subclades also exist. In contrast, in the second non-LTR tree (panel d), *Atline1a* (RU152; 161 and 110 copies in *A. thaliana* and *A. lyrata*, respectively), elements from *A. thaliana* and *A. lyrata* are more clearly separated. Only very few copies of *A. thaliana* are present within subclades of *A. lyrata* and vice versa.

The bottom row shows two examples of DNA elements (panels e and f). The left tree (e) contains more copies in *A. thaliana* (267 copies) than in *A. lyrata* (198 copies). The tree shows the *Atrep3* family (RU287), which is the most abundant family in *A. thaliana*. The tree shows many recent copies in *A. thaliana* (indicated by short branch lengths), which all group together in a few closely related subclades. These subclades also contain some more diverged elements, and only one copy from *A. lyrata*. The copies of *A. lyrata* are older in general and separate into different subclades. Some more diverged copies from *A. thaliana* are also spread throughout these clades. The second DNA family (panel f), *Atrep9* (RU293), shows a different phylogenetic distribution. Here, copies in the two species are clearly separated. All ten *A. thaliana* copies form one subclade that is distantly related to all copies in *A. lyrata*. The elements in *A. lyrata* are quite diverged. Many copies form their own subclade and only a few cluster together.

Conservative analysis

The de novo identification of repeat families has a serious disadvantage: it often returns several sequence fragments for one TE family instead of one full length transposable element. One family might therefore be represented by several shorter sequences, which might influence our analysis. We therefore created a second element library containing only RepeatScout sequences with a length of at least 2,000 bp and all Repbase Update sequences. We repeated our analysis with this "conservative" library and overall find similar results than in the full family set.

Our conservative library consisted of 357 sequences from Repbase Update and 243 de novo families identified by RepeatScout resulting in a total of 600 families. We identified more than 63,000 copies in both genomes. We find that *A. lyrata* contains with more than 47,000 copies almost three times more copies than *A. thaliana* with 16,000 copies. This result also holds for each TE class seperately (see Additional Figure S11). The higher copy number in *A. lyrata* is in agreement with our full family set. The TE copies in *A. thaliana* represent around 16 Mbps of the genome sequence (13 percent) and in *A. lyrata* around 43 Mbps (21 percent). We again find a higher TE copy number per Mbps in *A. lyrata* (228 copies per Mbps) than in *A. thaliana* (140 copies per Mbps). In both species the majority of elements are DNA elements (60 percent), followed by LTR elements (27 percent), non-LTR elements (12 percent) and unclassified elements (1 percent). The distribution of copies in TE classes is the same as for our complete element set, only the percentage varies slightly.

Out of our 600 families we find that 550 families are shared between both species. The remaining 50 families are unique to either one species. As for our full family set, we find a much higher number of unique families in *A. lyrata* (43 families) than in *A. thaliana* (7 families). If we compare the copy number distribution between the shared and unique families (see Additional Figure S12) we find a

significantly higher copy number for shared families in *A. lyrata* ($p < 10^{-15}$, Wilcoxon rank sum test). The average copy number per family is with 15 copies much lower in *A. thaliana* than in *A. lyrata* with 43 copies.

If we compare the average insertion time for TE families in both genomes (see Additional Figure S13), we find slightly more recent average insertion times in *A. lyrata* with 11.9 Mya than in *A. thaliana* with 12.9 Mya. As in agreement with the results from our full family set, we find a roughly exponential age distribution for *A. lyrata* elements (see Additional Figure S14, light grey bars). In contrast, the age distribution for *A. thaliana* has a significant longer mean element age than elements in *A. lyrata* ($p < 10^{-15}$, Wilcoxon rank sum test). Similar to the results of the full family set, we find a drop in element abundance for elements younger than 500,000 years old (see Additional Figure S14, dark grey bars), the approximate time range when self-incompatibility was lost in *A. thaliana*.

In sum, the conservative library yields similar conclusions as the full family set, making it unlikely that family fragmentation by our de novo approach influences our results.

Additional Figures

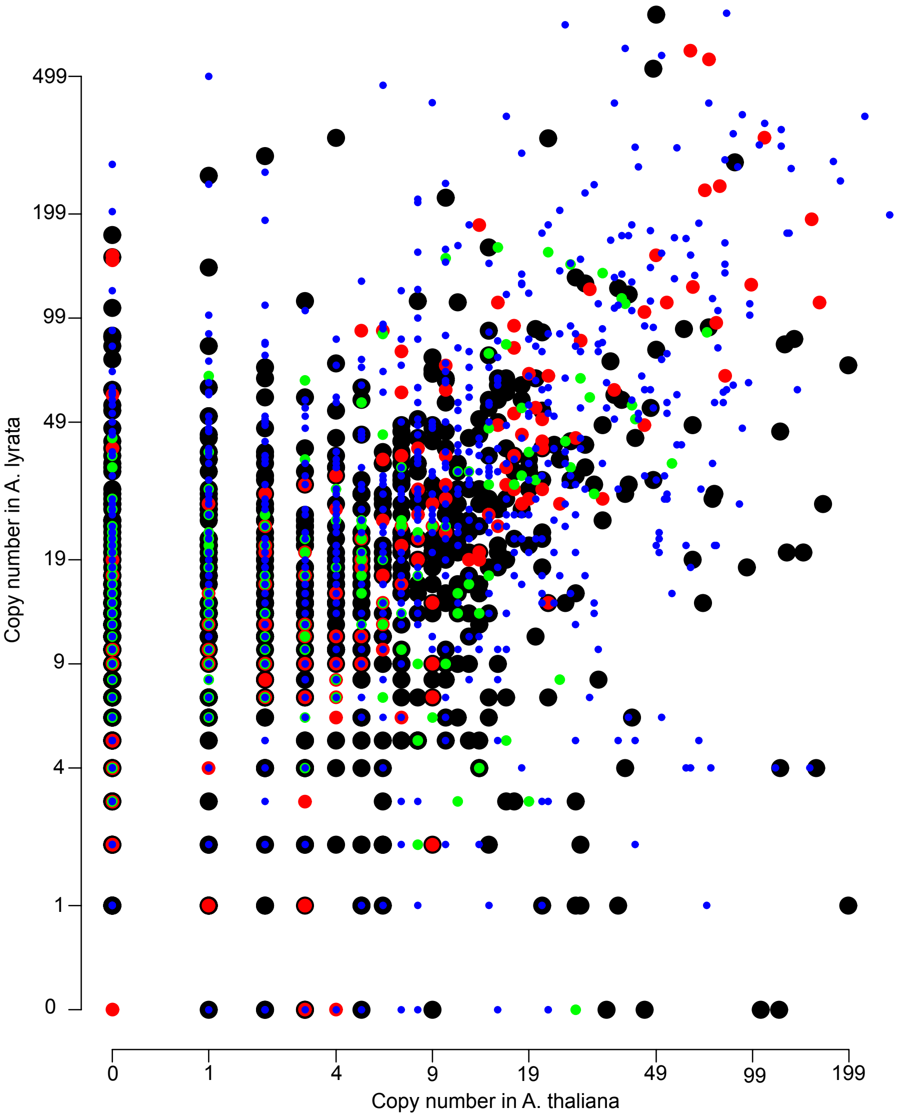

Figure S1 Relationship between copy numbers in *A. thaliana* and *A. lyrata* for each family. The figure shows data for different TE classes indicated by black, red, green and blue points for LTR, non-LTR, DNA and unclassified families, respectively. This figure corresponds to Figure 1 but the data is represented on a log scale.

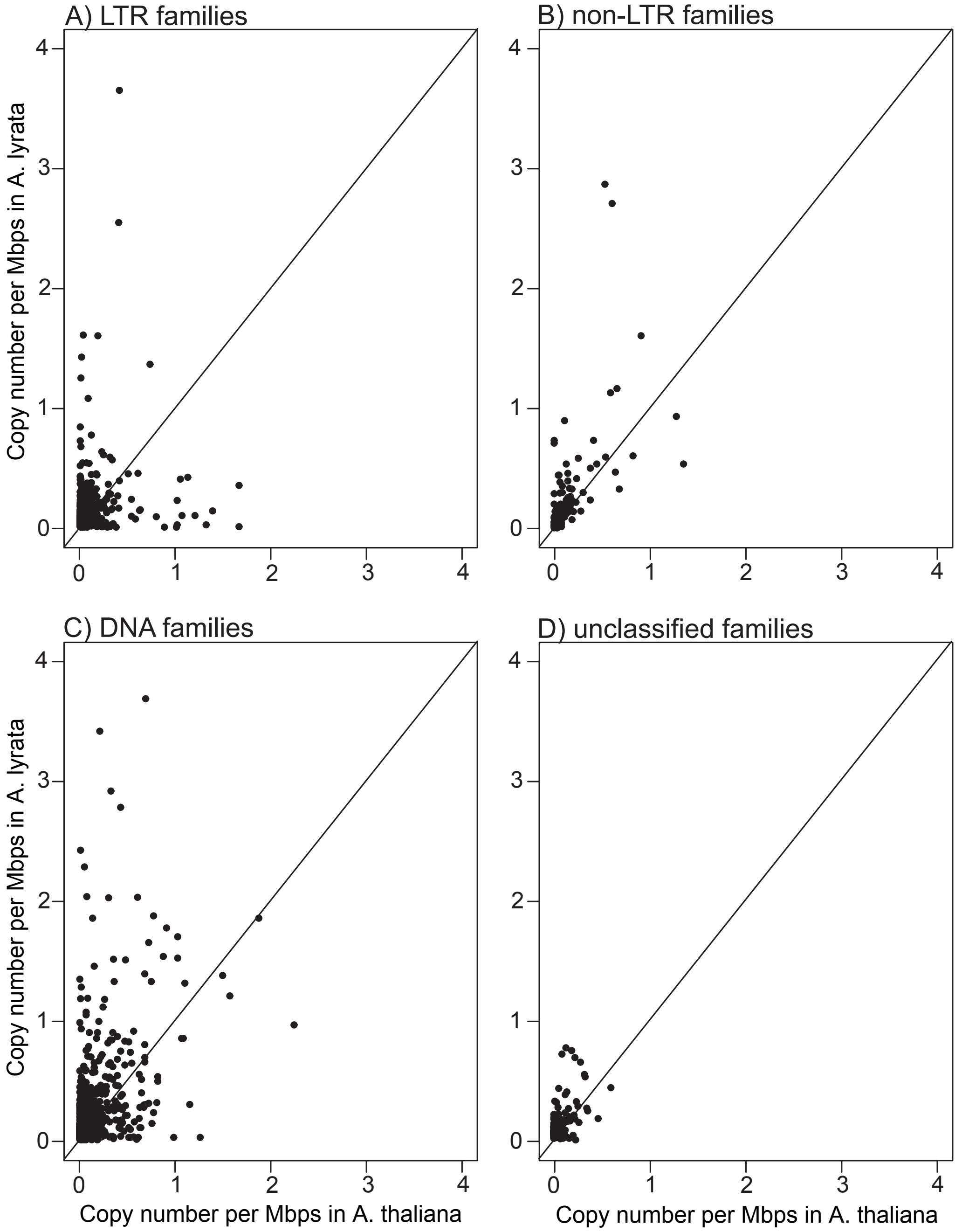

Figure S2 Relationship between copy numbers per million base pairs (Mbps) in *A. thaliana* and *A. lyrata* for each family. The panels show data for different TE classes, as indicated above each panel. The diagonal black line represents the line of equal copy numbers per Mbps in both species. Families with a higher copy number in *A. thaliana* and *A. lyrata* thus correspond to points below and above the diagonal line, respectively.

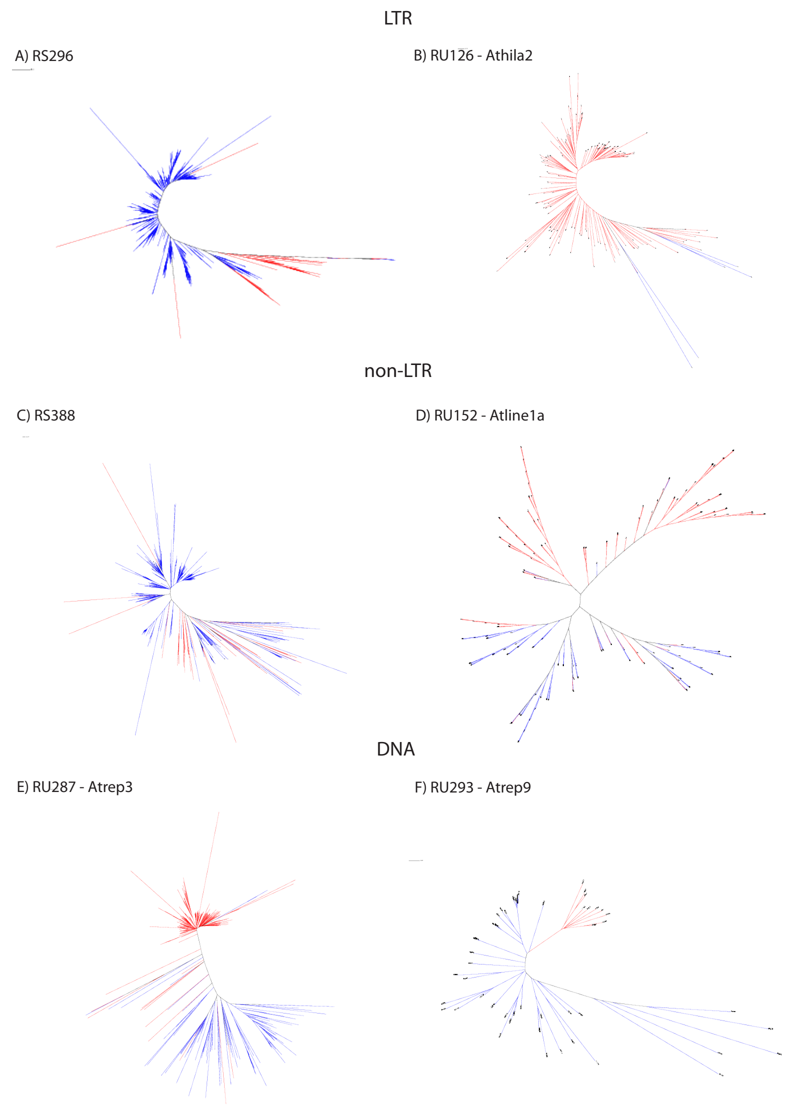

Figure S3 Phylogenetic trees based on nucleotide alignments for six representative families. Based on a multiple alignment of all sequences longer than 200 bp a phylogenetic tree was constructed (see Methods). Red branches indicate a copy from *A. thaliana* and blue branches a copy in *A. lyrata*. The length of a branch indicates the divergence of the element, with a higher divergence represented by a longer branch.

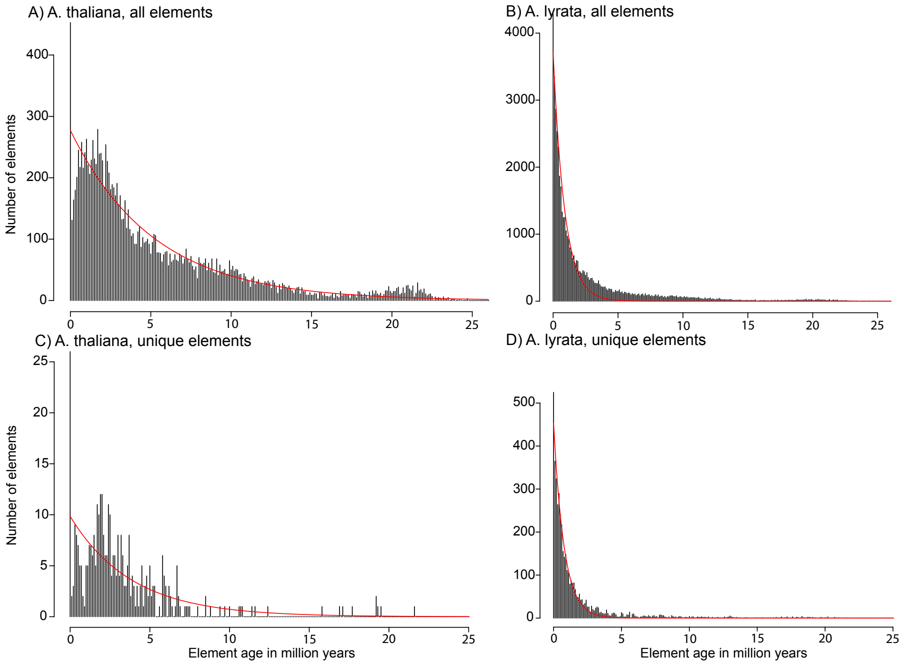

Figure S4 Insertion time distribution for all elements, fitted to an exponential distribution. The figure shows the total number of elements (vertical axis) with a given insertion time (horizontal axis) for A) all elements in *A. thaliana*, B) all elements in *A. lyrata*, C) elements in families unique to *A. thaliana* and D) elements in families unique to *A. lyrata*. The red curve in each figure represents a fit to an exponential function, as estimated based on a nonlinear least squares method. Note the especially poor fit in a). For c) only 363 elements were used which makes an estimation difficult.

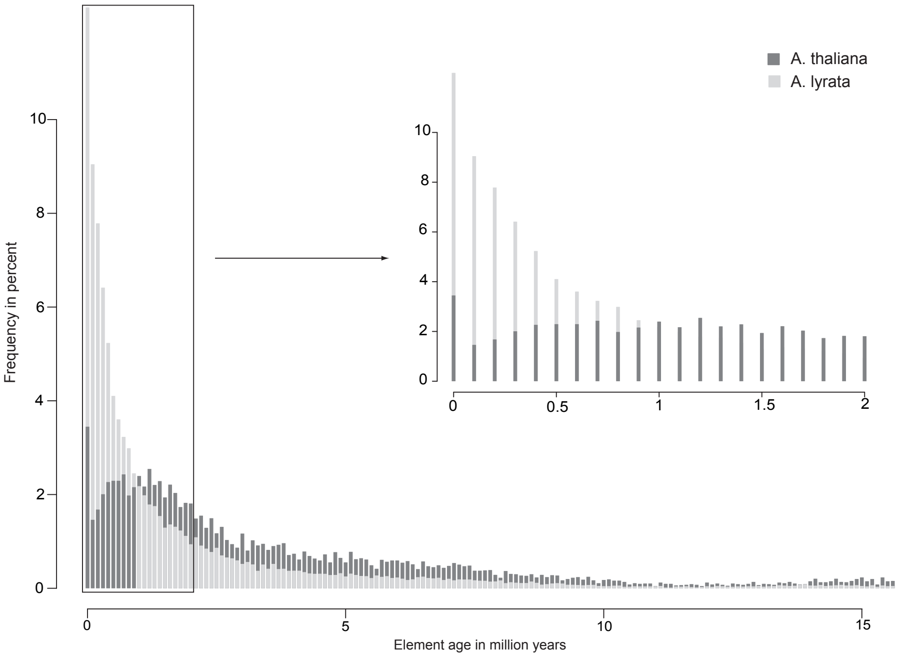

Figure S5 Insertion time distribution for all elements based on a substitution rate of 0.021 per site per million years. A histogram of the number of elements with a given insertion time (horizontal axis). Values on the vertical axis are given as percent of the total number of elements. Elements in *A. thaliana* are represented by dark grey bars, and elements in *A. lyrata* by light grey bars. For visual clarity, the figure only shows elements with an insertion time less than 15 million years ago. Only few elements were inserted even earlier. The inset shows the frequency of elements younger than 2 million years. We note that for each copy number value on the horizontal axis, the bars for both *A. thaliana* and *A. lyrata* originate at a value of zero on the vertical axis. Thus, bars are not stacked, despite their visual appearance, and do not represent the sum of copies in both species. We used this format to ensure visual clarity despite the large number of bars in each histogram.

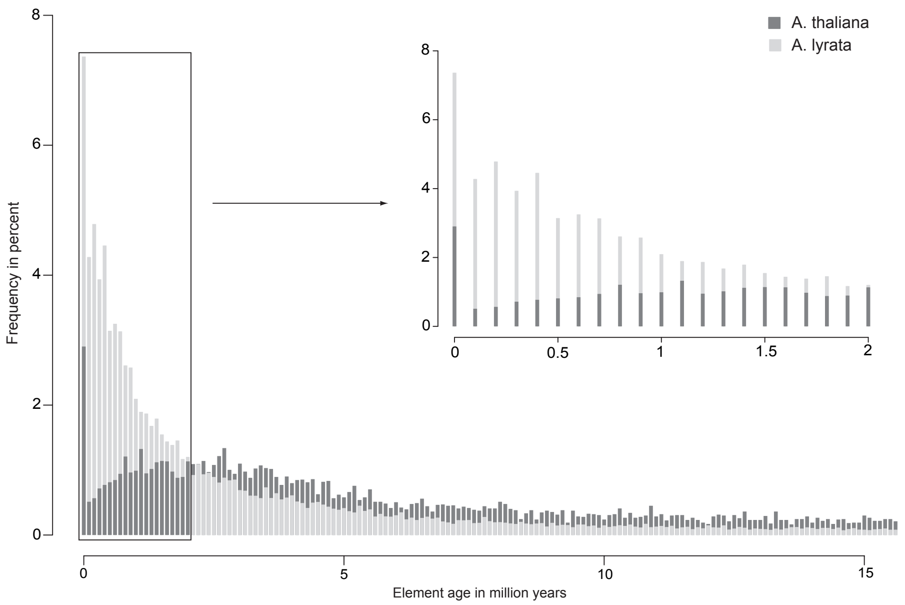

Figure S6 Insertion time distribution for all elements based on a substitution rate of 0.0099 per site per million years. A histogram of the number of elements with a given insertion time (horizontal axis). Values on the vertical axis are given as percent of the total number of elements. Elements in *A. thaliana* are represented by dark grey bars, and elements in *A. lyrata* by light grey bars. For visual clarity, the figure only shows elements with an insertion time less than 15 million years ago. Only few elements were inserted even earlier. The inset shows the frequency of elements younger than 2 million years. We note that for each copy number value on the horizontal axis, the bars for both *A. thaliana* and *A. lyrata* originate at a value of zero on the vertical axis. Thus, bars are not stacked, despite their visual appearance, and do not represent the sum of copies in both species. We used this format to ensure visual clarity despite the large number of bars in each histogram.

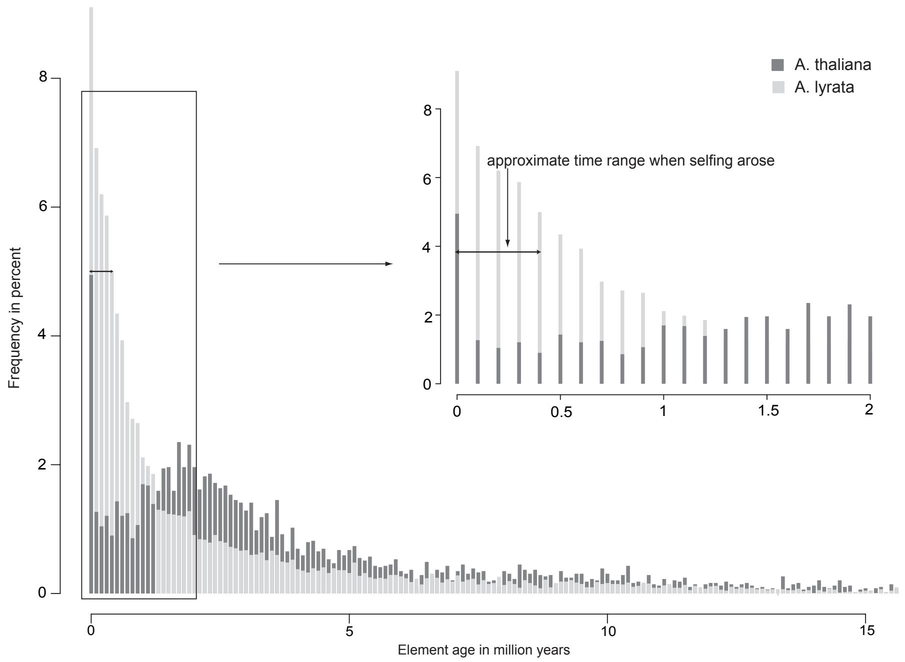

Figure S7 Insertion time distribution for all elements belonging to LTR families. A histogram of the number of LTR elements with a given insertion time (horizontal axis). Values on the vertical axis are given as percent of the total number of elements. Elements in *A. thaliana* are represented by dark grey bars, and elements in *A. lyrata* by light grey bars. For visual clarity, the figure only shows elements with an insertion time less than 15 million years ago. Only few elements were inserted even earlier. The inset shows the frequency of elements younger than 2 million years. The double-headed arrow indicates the approximate time when selfing arose in *A. thaliana* [10, 11]. We note that for each copy number value on the horizontal axis, the bars for both *A. thaliana* and *A. lyrata* originate at a value of zero on the vertical axis. Thus, bars are not stacked, despite their visual appearance, and do not represent the sum of copies in both species. We used this format to ensure visual clarity despite the large number of bars in each histogram.

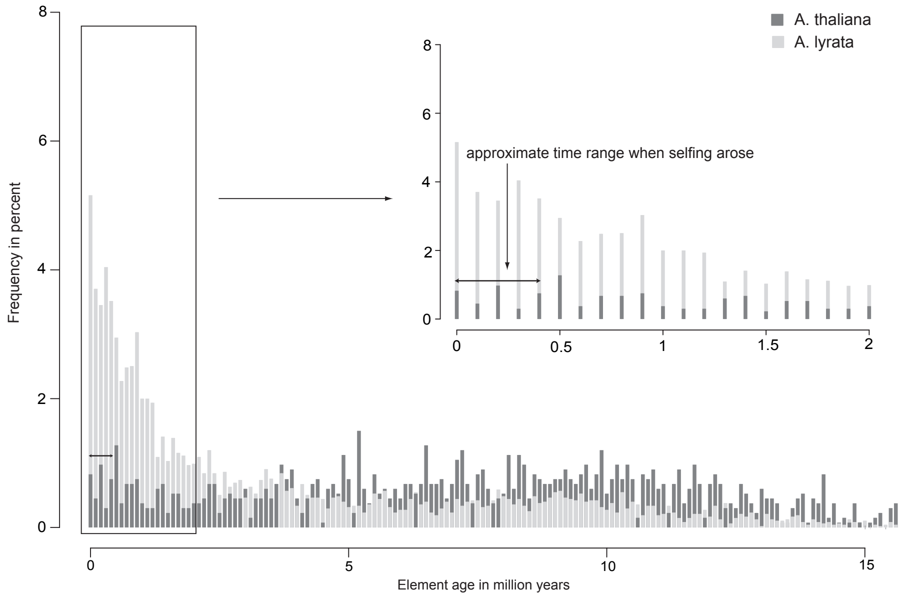

Figure S8 Insertion time distribution for all elements belonging to non-LTR families. A histogram of the number of non-LTR elements with a given insertion time (horizontal axis). Values on the vertical axis are given as percent of the total number of elements. Elements in *A. thaliana* are represented by dark grey bars, and elements in *A. lyrata* by light grey bars. For visual clarity, the figure only shows elements with an insertion time less than 15 million years ago. Only few elements were inserted even earlier. The inset shows the frequency of elements younger than 2 million years. The double-headed arrow indicates the approximate time when selfing arose in *A. thaliana* [10, 11]. We note that for each copy number value on the horizontal axis, the bars for both *A. thaliana* and *A. lyrata* originate at a value of zero on the vertical axis. Thus, bars are not stacked, despite their visual appearance, and do not represent the sum of copies in both species. We used this format to ensure visual clarity despite the large number of bars in each histogram.

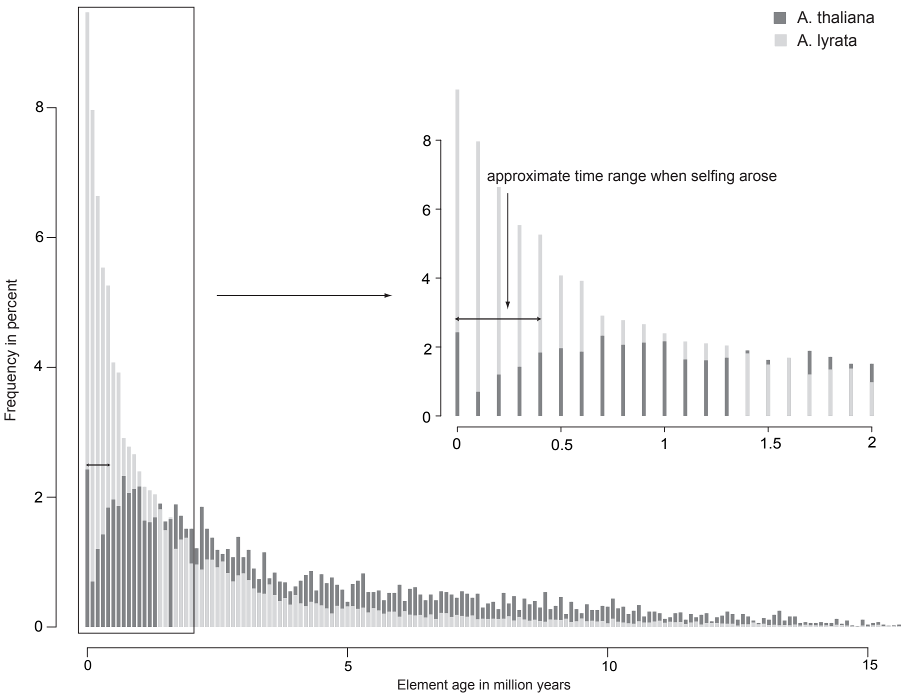

Figure S9 Insertion time distribution for all elements belonging to DNA families. A histogram of the number of DNA elements with a given insertion time (horizontal axis). Values on the vertical axis are given as percent of the total number of elements. Elements in *A. thaliana* are represented by dark grey bars, and elements in *A. lyrata* by light grey bars. For visual clarity, the figure only shows elements with an insertion time less than 15 million years ago. Only few elements were inserted even earlier. The inset shows the frequency of elements younger than 2 million years. The double-headed arrow indicates the approximate time when selfing arose in *A. thaliana* [10, 11]. We note that for each copy number value on the horizontal axis, the bars for both *A. thaliana* and *A. lyrata* originate at a value of zero on the vertical axis. Thus, bars are not stacked, despite their visual appearance, and do not represent the sum of copies in both species. We used this format to ensure visual clarity despite the large number of bars in each histogram.

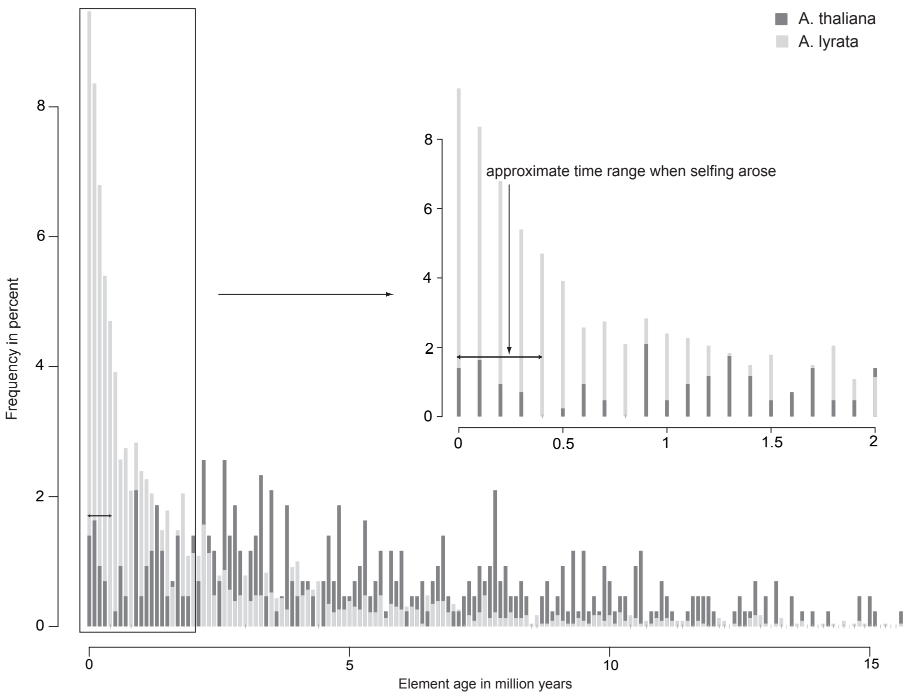

Figure S10 Insertion time distribution for elements belonging to unclassified families. A histogram of the number of unclassified elements with a given insertion time (horizontal axis). Values on the vertical axis are given as percent of the total number of elements. Elements in *A. thaliana* are represented by dark grey bars, and elements in *A. lyrata* by light grey bars. For visual clarity, the figure only shows elements with an insertion time less than 15 million years ago. Only few elements were inserted even earlier. The inset shows the frequency of elements younger than 2 million years. The double-headed arrow indicates the approximate time when selfing arose in *A. thaliana* [10, 11]. We note that for each copy number value on the horizontal axis, the bars for both *A. thaliana* and *A. lyrata* originate at a value of zero on the vertical axis. Thus, bars are not stacked, despite their visual appearance, and do not represent the sum of copies in both species. We used this format to ensure visual clarity despite the large number of bars in each histogram.

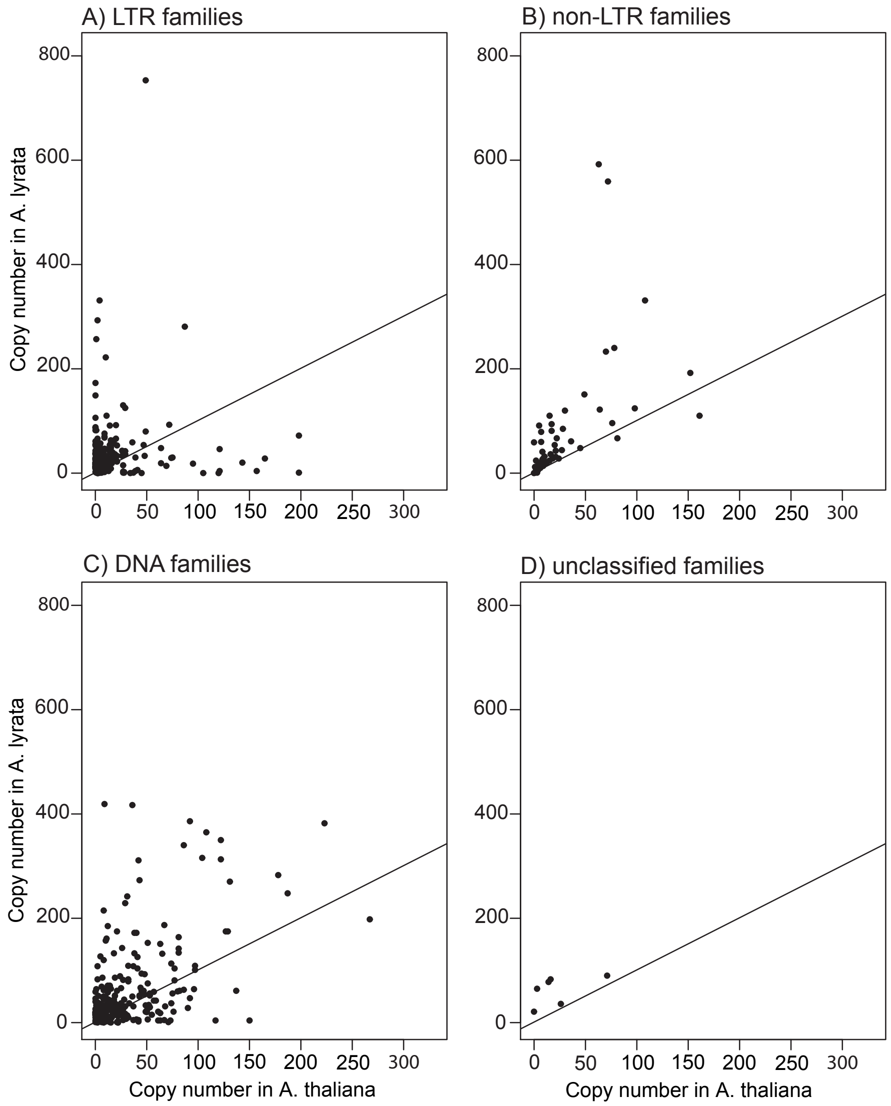

Figure S11 Relationship between copy numbers in *A. thaliana* and *A. lyrata* for each family of the conservative set. The panels show data for different TE classes, as indicated above each panel. The diagonal black line represents the line of equal copy numbers in both species. Families with a higher copy number in *A. thaliana* and *A. lyrata* thus correspond to points below and above the diagonal line, respectively.

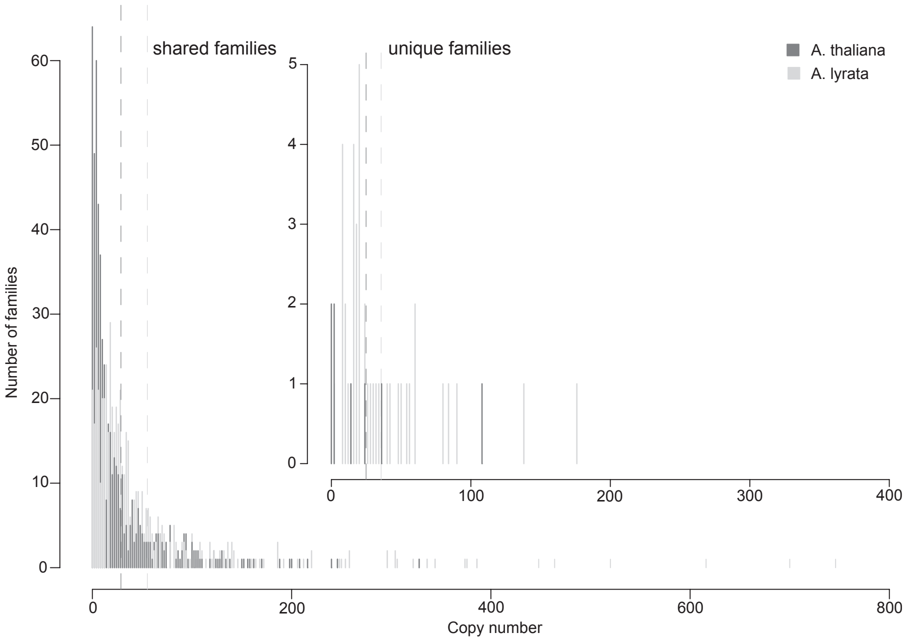

Figure S12 Copy number distribution of TEs from the conservative set in both genomes. The figure shows a histogram of element copy number (horizontal axis) divided into families shared between the two genomes and unique families (inset). Dark grey bars represent the number of families in *A. thaliana* and light grey bars in *A. lyrata*. The dashed lines of the same shading indicate the average copy number per family for the respective species. We note that for each copy number value on the horizontal axis, the bars for both *A. thaliana* and *A. lyrata* originate at a value of zero on the vertical axis. Thus, bars are not stacked, despite their visual appearance, and do not represent the sum of copies in both species. We used this format to ensure visual clarity despite the large number of bars in each histogram. Notice the different vertical scales for shared and unique families.

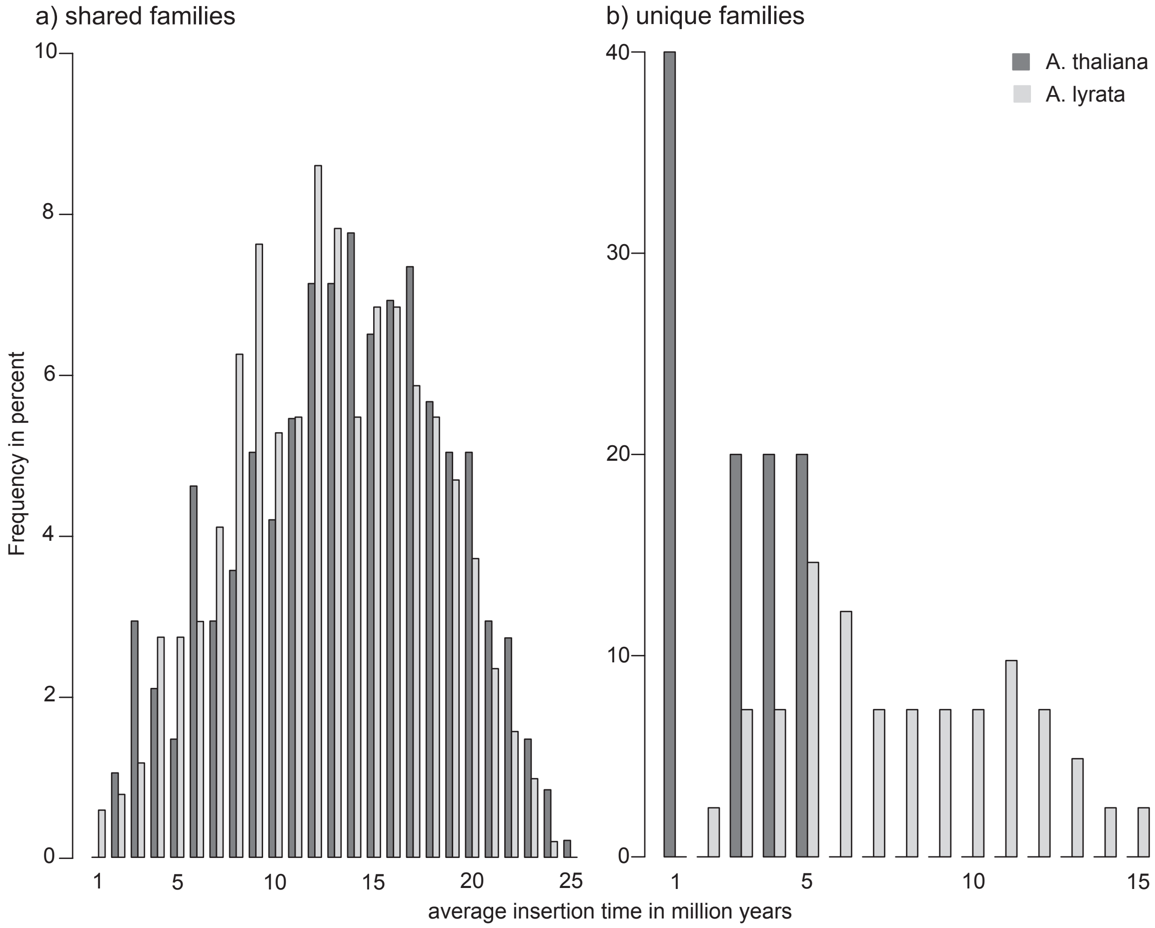

Figure S13 Average insertion time distribution for each family of the conservative set. The average insertion time was calculated based on the average nucleotide divergence between all copies of one family. A) histogram for shared families; B) histogram for unique families. The percentage of families with a given age are represented by dark grey bars for *A. thaliana* and light grey bars for *A. lyrata*. The mean average insertion time for shared families is 13 Mya (million years ago) for *A. thaliana* and 12.2 Mya for *A. lyrata*, respectively. For unique families it is 2.4 Mya and 7.3 Mya, respectively. The mean of the average insertion time for shared families is significantly smaller in *A. lyrata* ($p < 0.01$, Wilcoxon rank sum test). Notice the different scales for shared and unique families

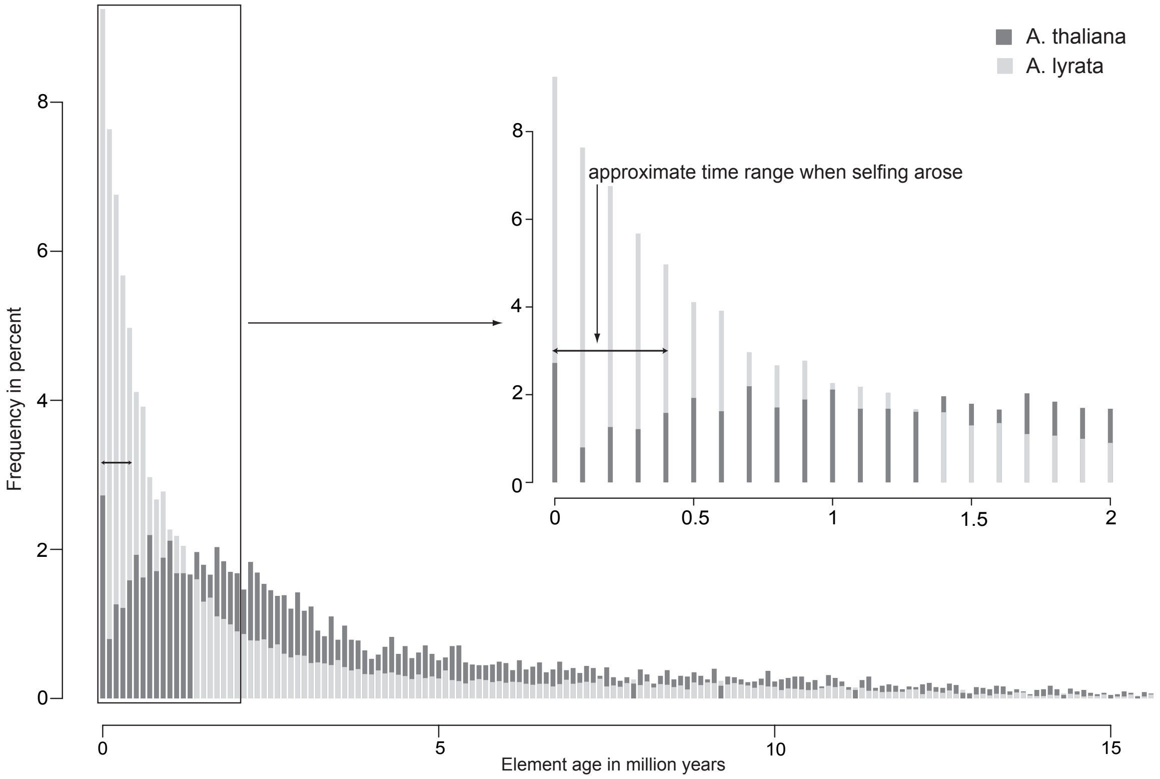

Figure S14 Insertion time distribution for all elements form the conservative set. A histogram of the number of elements with a given insertion time (horizontal axis). Values on the vertical axis are given as percent of the total number of elements. Elements in *A. thaliana* are represented by dark grey bars, and elements in *A. lyrata* by light grey bars. For visual clarity, the figure only shows elements with an insertion time less than 15 million years ago. Only few elements were inserted even earlier. The inset shows the frequency of elements younger than 2 million years. The double-headed arrow indicates the approximate time range when selfing arose in *A. thaliana* [10, 11]. We note that for each copy number value on the horizontal axis, the bars for both *A. thaliana* and *A. lyrata* originate at a value of zero on the vertical axis. Thus, bars are not stacked, despite their visual appearance, and do not represent the sum of copies in both species. We used this format to ensure visual clarity despite the large number of bars in each histogram.

Additional Tables

		number families	average copy number	max copy number
DNA	shared *A thaliana*	660	19.05	267
	shared *A. lyrata*	660	56.0	760
	unique *A thaliana*	12	7.8	22
	unique *A. lyrata*	150	21.0	277
LTR	shared *A thaliana*	536	12.0	198
	shared *A. lyrata*	536	29.7	753
	unique *A thaliana*	11	29.9	120
	unique *A. lyrata*	131	20.0	173
non-LTR	shared *A thaliana*	126	17.9	161
	shared *A. lyrata*	126	50.9	592
	unique *A thaliana*	2	3.5	4
	unique *A. lyrata*	15	32.2	151
unclassified	shared *A thaliana*	126	9.3	71
	shared *A. lyrata*	126	29.2	159
	unique *A thaliana*	1	27	27
	unique *A. lyrata*	49	15.2	44

Table S2 Family number, average copy number, and maximal copy number for each TE class subdivided in shared and unique families

		Wright (2000)		Lockton (2010)		our results	
name	corresp. id	Atha	Alyr	Atha	Alyr	Atha	Alyr
gypsy-like	RU126/RU136			17.5	15.9	157/121	4/4
LINE-like	RU152			13.9	11.8	161	110
SINE-like	RU169			25.6	20.4	152	192
Ac-like	RU240	10.7	8.2	12.5	22	26	143
CACTA-like	RU219			10.1	7.6	10	28

Table S3 Copy numbers of TEs in *A. thaliana* (Atha) and *A. lyrata* (Alyr) in previous work compared to our observations.

References

[1] Alkes L. Price, Neil C. Jones, and Pavel A. Pevzner. De novo identification of repeat families in large genomes. *Bioinformatics*, 21 Suppl 1:i351–i358, Jun 2005.

[2] John C. Wooton and Scott Federhen. Statistics of local complexity in amino acid sequences and sequence databases. *Computers and Chemnistry*, 17:149–163, 1993.

[3] Gary Benson. Tandem repeats finder: a program to analyze DNA sequences. *Nucleic Acids Res*, 27(2):573–580, Jan 1999.

[4] Arian F. Smit, Robert Hubley, and Phil Green. RepeatMasker Open–3.0. http://repeatmasker.org, 1996–2010.

[5] Christoph Mayer. Phobos 3.3.11. http://www.rub.de/spezzoo/cm/cm_phobos.htm, 2006–2010.

[6] Cédric Feschotte, Umeshkumar Keswani, Nirmal Ranganathan, Marcel L. Guibotsy, and David Levine. Exploring repetitive DNA landscapes using REPCLASS, a tool that automates the classification of transposable elements in eukaryotic genomes. *Genome Biology and Evolution*, 1(1):205–220, 2009.

[7] György Abrusán, Norbert Grundmann, Luc DeMester, and Wojciech Makalowski. TEclass – a tool for automated classification of unknown eukaryotic transposable elements. *Bioinformatics*, 25(10):1329–1330, May 2009.

[8] Steven Lockton, Jeffrey Ross-Ibarra, and Brandon S. Gaut. Demography and weak selection drive patterns of transposable element diversity in natural populations of *Arabidopsis lyrata*. *Proc Natl Acad Sci U S A*, 105(37):13965–13970, Sep 2008.

[9] Steven Lockton and Brandon S. Gaut. The evolution of transposable elements in natural populations of self-fertilizing *Arabidopsis thaliana* and its outcrossing relative *Arabidopsis lyrata*. *BMC Evol Biol*, 10:10, 2010.

[10] Jesper S. Bechsgaard, Vincent Castric, Deborah Charlesworth, Xavier Vekemans, and Mikkel H. Schierup. The transition to self-compatibility in *Arabidopsis thaliana* and evolution within S-haplotypes over 10 Myr. *Mol Biol Evol*, 23(9):1741–1750, Sep 2006.

[11] Takashi Tsuchimatsu, Keita Suwabe, Rie Shimizu-Inatsugi, Sachiyo Isokawa, Pavlos Pavlidis, Thomas Stdler, Go Suzuki, Seiji Takayama, Masao Watanabe, and Kentaro K Shimizu. Evolution of self-compatibility in *Arabidopsis* by a mutation in the male specificity gene. *Nature*, 464(7293):1342–1346, Apr 2010.
